# The Bacteriophage–Phage-Inducible Chromosomal Island Arms Race Designs an Interkingdom Inhibitor of dUTPases

**DOI:** 10.1128/spectrum.03232-22

**Published:** 2023-01-09

**Authors:** Carla Sanz-Frasquet, J. Rafael Ciges-Tomas, Christian Alite, José R. Penadés, Alberto Marina

**Affiliations:** a Instituto de Biomedicina de Valencia (IBV), CSIC and CIBER de Enfermedades Raras (CIBERER), Valencia, Spain; b Institute of Infection, Immunity and Inflammation, College of Medical, Veterinary and Life Sciences, University of Glasgow, Glasgow, United Kingdom; c MRC Centre for Molecular Bacteriology and Infection, Imperial College London, London, United Kingdom; Centre national de la recherche scientifique, Aix-Marseille Université

**Keywords:** dUTPase, inhibition, Stl repressor, crystal structure, protein-protein interaction, Dut-Stl complex, PICI

## Abstract

Stl, the master repressor of the Staphylococcus aureus pathogenicity islands (SaPIs), targets phage-encoded proteins to derepress and synchronize the SaPI and the helper phage life cycles. To activate their cycle, some SaPI Stls target both phage dimeric and phage trimeric dUTPases (Duts) as antirepressors, which are structurally unrelated proteins that perform identical functions for the phage. This intimate link between the SaPI’s repressor and the phage inducer has imposed an evolutionary optimization of Stl that allows the interaction with Duts from unrelated organisms. In this work, we structurally characterize this sophisticated mechanism of specialization by solving the structure of the prototypical SaPIbov1 Stl in complex with a prokaryotic and a eukaryotic trimeric Dut. The heterocomplexes with Mycobacterium tuberculosis and Homo sapiens Duts show the molecular strategy of Stl to target trimeric Duts from different kingdoms. Our structural results confirm the participation of the five catalytic motifs of trimeric Duts in Stl binding, including the C-terminal flexible motif V that increases the affinity by embracing Stl. *In silico* and *in vitro* analyses with a monomeric Dut support the capacity of Stl to recognize this third family of Duts, confirming this protein as a universal Dut inhibitor in the different kingdoms of life.

**IMPORTANCE** Stl, the Staphylococcus aureus pathogenicity island (SaPI) master repressor, targets phage-encoded proteins to derepress and synchronize the SaPI and the helper phage life cycles. This fascinating phage-SaPI arms race is exemplified by the Stl from SaPIbov1 which targets phage dimeric and trimeric dUTPases (Duts), structurally unrelated proteins with identical functions in the phages. By solving the structure of the Stl in complex with a prokaryotic (M. tuberculosis) and a eukaryotic (human) trimeric Dut, we showed that Stl has developed a sophisticated substrate mimicry strategy to target trimeric Duts. Since all these Duts present identical catalytic mechanisms, Stl is able to interact with Duts from different kingdoms. In addition, *in silico* modeling with monomeric Dut supports the capacity of Stl to recognize this third family of Duts, confirming this protein as a universal Dut inhibitor.

## INTRODUCTION

Bacteria are the hosts for viruses and other mobile genetic elements (MGEs). In many cases this MGE-host relationship is detrimental to the latter; thus, the host cells have evolved multiple systems and mechanisms to defend against the MGEs. For their part, MGEs respond with countermeasures that block or evade these defenses, producing a true arms race that promotes the evolution of both contenders. However, this contest is even more complex, since some MGEs can also be parasitized by other MGEs, imposing complex tradeoff evolutionary processes in the “ménage à trois” generated by the MGEs and their host cell (1, 2). Satellite viruses and phage-inducible chromosomal islands (PICIs), together with their helper viruses and the corresponding host cells, exemplify this scenario. Both types of MGEs depend on helper viruses to complete their life cycle and, consequently, have evolved sophisticated mechanisms to synchronize with the parasitized virus (3). A fascinating example of this synchronization mechanism is typified by the Staphylococcus aureus pathogenicity islands (SaPIs), which are the prototypical PICIs (4). SaPIs reside passively in the bacterial host chromosome under the strict control of Stl, the master repressor encoded by the SaPI itself. Once the helper virus, a bacteriophage (phage) in this case, infects the host bacteria or a helper prophage is induced, the SaPI cycle is activated, producing phage-like particles composed of phage virion proteins that encapsulate the SaPI genome (5). In order to have an exquisite synchronization with the helper phage life cycle, the SaPI Stl repressor recognizes a specific protein expressed by the helper phage to release its DNA operator and to start up the SaPI cycle (3, 6, 7). Since SaPI induction imposes a high cost for the phage, phages try to overcome the problem by using alternative strategies such as the generation of allelic variants of the antirepressor with lower affinity for Stl (8) or, alternatively, replacing the antirepressor with another protein which has identical biological activity but is completely unrelated structurally (6). This strategy has been observed in phages that have substituted trimeric dUTPases (Duts) (all-beta folding), which were originally described as the antirepressors of SaPIbov1 (3), for dimeric Duts (all-alpha folding), which, although they catalyze the same reaction, are structurally and functionally different (9, 10). Surprisingly, both trimeric and dimeric Duts are able to derepress SaPIbov1 by interaction with Stl (6). This observation shows that SaPIs have gone a step further in the arms race and no longer target a specific protein but a basic biological process for the helper phages, preventing the escape or imposing an inadmissible cost for the phage. But how has the island managed to recognize a phage process and not a specific protein? Our previous work (11) has shown that the Stl repressor, an all-alpha helix protein, is a modular protein composed of an N-terminal domain with the characteristic helix-turn-helix (HTH) folding, which mediates DNA binding, followed by a middle and a C-terminal dimerization domain. The middle domain is specialized to recognize trimeric Duts, while the C-terminal domain recognizes dimeric Duts. Recently, the three-dimensional structures of the complexes of N- and C-terminal portions of SaPIbov1 Stl (here Stl) with trimeric and dimeric Duts from phages ϕ11 and ϕΟ11, respectively, were solved, showing a mimicking strategy used by the repressor to target a biological process of the phage (11).

The structure of phage ϕ11 trimeric Dut (ϕ11Dut) in complex with the N-terminal portion (N-terminal and middle domains) of Stl showed three independent Stl monomers that bind to the three active centers of the Dut trimer, interacting with catalytic residues from 4 of the 5 conserved motifs in the trimeric Duts. Therefore, Stl follows a mimicry mechanism to recognize the trimeric Dut by emulating dUTP substrate interactions. Similarly, the C-terminal domain of Stl also follows this strategy to recognize dimeric Duts, since the structure of the phage O11 dimeric Dut in complex with this Stl portion showed interactions with conserved catalytic residues of the dimeric Dut. By mimicking the substrate dUTP, Stl targets a phage process, thus preventing escape even if the enzyme used by the phage to carry out the process is changed. As the recognition of the substrate dUTP and its mechanism of hydrolysis is different for dimeric and trimeric Duts, Stl has had to recruit two independent domains in order to recognize both types of enzymes, confirming the high degree of evolution acquired by this protein to avoid phage evasion. Trimeric Duts show high sequence conservation at the residues that make up their active centers, which has been used to define five catalytic motifs (motifs I to V) (see Fig. S1 in the supplemental material) that are the signature of this family of enzymes (12). The exquisite dUTP mimicry observed may indicate that Stl would have a broad spectrum of interactions with trimeric Duts. This seems to be the case, since Stl interaction with the Mycobacterium tuberculosis, Drosophila melanogaster, and Homo sapiens Duts has been reported, and Stl has been proposed as a proteinaceous inhibitor of trimeric Duts (13–15). Although the *in vitro* characterization of the interaction of Stl with these prokaryotic and eukaryotic trimeric Duts suggests a similar molecular mechanism of recognition, some discrepancies have been observed—for example, while equimolecular interactions (one Dut trimer with three Stls) similar to the ϕ11Dut-Stl structure have been reported, alternative stoichiometries (one Dut trimer interacting with one Stl dimer or 2 Stl monomers) have also been proposed (15).

In this work, we take a step forward in the study of the molecular basis of this fascinating mechanism of targeting biological processes used by the main SaPIbov1 repressor. Here, we solve the structure of the Stl N-terminal portion in complex with prokaryotic (M. tuberculosis) and eukaryotic (H. sapiens) trimeric Duts. The structures confirm the exquisite molecular mimicry mechanism used for the Stl to recognize Duts from different kingdoms of life. Moreover, the structure shows how motif V of the human Dut, a P-loop that covers the active center once the substrate has been bound, also participates in the interactions. Therefore, Stl targets all the catalytic motifs of the trimeric Duts and thereby reduces its escape capacity. Moreover, our *in silico* and *in vitro* studies show that Stl would also be able to recognize monomeric Duts, thus confirming this protein as a universal Dut inhibitor.

## RESULTS

### The N-terminal portion of Stl binds to Mycobacterium tuberculosis and human Duts.

To better understand the molecular basis of the broad-spectrum inhibitory capacity on trimeric Duts shown by the Stl repressor from SaPIbov1 (13, 15, 16), we undertook the structural characterization of this repressor in complex with one representative prokaryotic and one representative eukaryotic Dut. We selected the Duts from M. tuberculosis (mDut) and humans (hDut) since their interaction with Stl had been previously demonstrated (14, 15, 17). Functional and structural analyses have shown that Stl is a modular protein with a highly flexible region between its middle and C-terminal domains which, respectively, mediate the recognition and interaction with trimeric and dimeric Duts from S. aureus phages (11). This flexibility has hampered obtaining the three-dimensional structure of the full-length Stl, both alone and in complex with its target Duts. Therefore, for our structural studies we decided to use a construct that included the Stl N-terminal DNA-binding domain (DBD) and the middle domain (Stl^N-ter^; residues 1 to 156), which had already been used to obtain the structure in complex with the trimeric Dut of S. aureus phage ϕ11 (ϕ11Dut) (11). As a first step, we analyzed whether this portion of Stl is equally responsible for recognizing prokaryotic and eukaryotic trimeric Duts (6, 14, 15). Native PAGE assays showed that for both mDut and hDut, a band corresponding to the Dut-Stl^N-ter^ complex appears concomitantly with the disappearance of the bands corresponding to each individual protein (see Fig. S2 in the supplemental material). Titration assays showed that at a molar ratio of 1:1, the formation of the Dut-Stl^N-ter^ complex is maximal with a minimal residual amount of each of the participating proteins, a pattern identical to that observed for the control protein ϕ11Dut. These assays support the interaction model observed in the ϕ11Dut-Stl^N-ter^ complex structure, in which a trimer of ϕ11Dut binds three independent Stl^N-ter^ monomers (11). This equimolar stoichiometry has also been proposed for the complex of full-length Stl with mDut and hDut using alternative experimental approaches (14, 15). As a second step, we analyzed the binding kinetics of Stl^N-ter^ for these Duts using biolayer interferometry (BLI). Our analysis showed that the two Duts have almost identical affinities for Stl^N-ter^ (*K_D_* [equilibrium dissociation constant], 34.44 and 39.25 nM for hDut and mDut, respectively), and that this is only 5 times lower than that shown for ϕ11Dut (7.94 nM), one of its biological targets (Table 1; Fig. S3). Similar differences in affinity between ϕ11Dut and hDut for the full-length Stl were reported previously using the isothermal titration calorimetry (ITC) technique (0.23 and 0.10 μM for hDut and ϕ11Dut, respectively) (15, 18). These differences in affinity in relation to ϕ11Dut are mainly due to a higher dissociation rate constant for hDut and mDut (around 10- and 5-times-higher *K*_off_, respectively), since both Duts showed a similar (mDut) or even higher (hDut) association rate constant (Table 1; Fig. S3). Furthermore, hDut and mDut showed affinities for Stl^N-ter^ similar to that of the Dut from S. aureus phage 80α (40 nM [19]). These results support that this portion of Stl has selectively evolved to recognize trimeric enzymes that catalyze dUTP hydrolysis.

**TABLE 1:** Kinetic parameters of Stl^N-ter^ binding to trimeric Duts determined by BLI

Dut	*K_D_* (nM)	*K*_on_ (M^−1^ s^−1^)	*K*_off_ (s^−1^)	*R* ^2^ ^*a*^
mDut	39.25	2.56 × 10^5^	10.00 × 10^−3^	0.990
mDut H145F	12.46	3.88 × 10^5^	4.83 × 10^−3^	0.992
hDut	34.44	5.83 × 10^5^	20.20 × 10^−3^	0.974
ϕ11Dut	7.94	2.84 × 10^5^	2.25 × 10^−3^	0.990

a *R*^2^ value indicates accuracy between fit and experimental data. Values above 0.95 are considered a good fit.

### Structures of Stl^N-ter^ bound to mDut and hDut.

**FIG 1:**
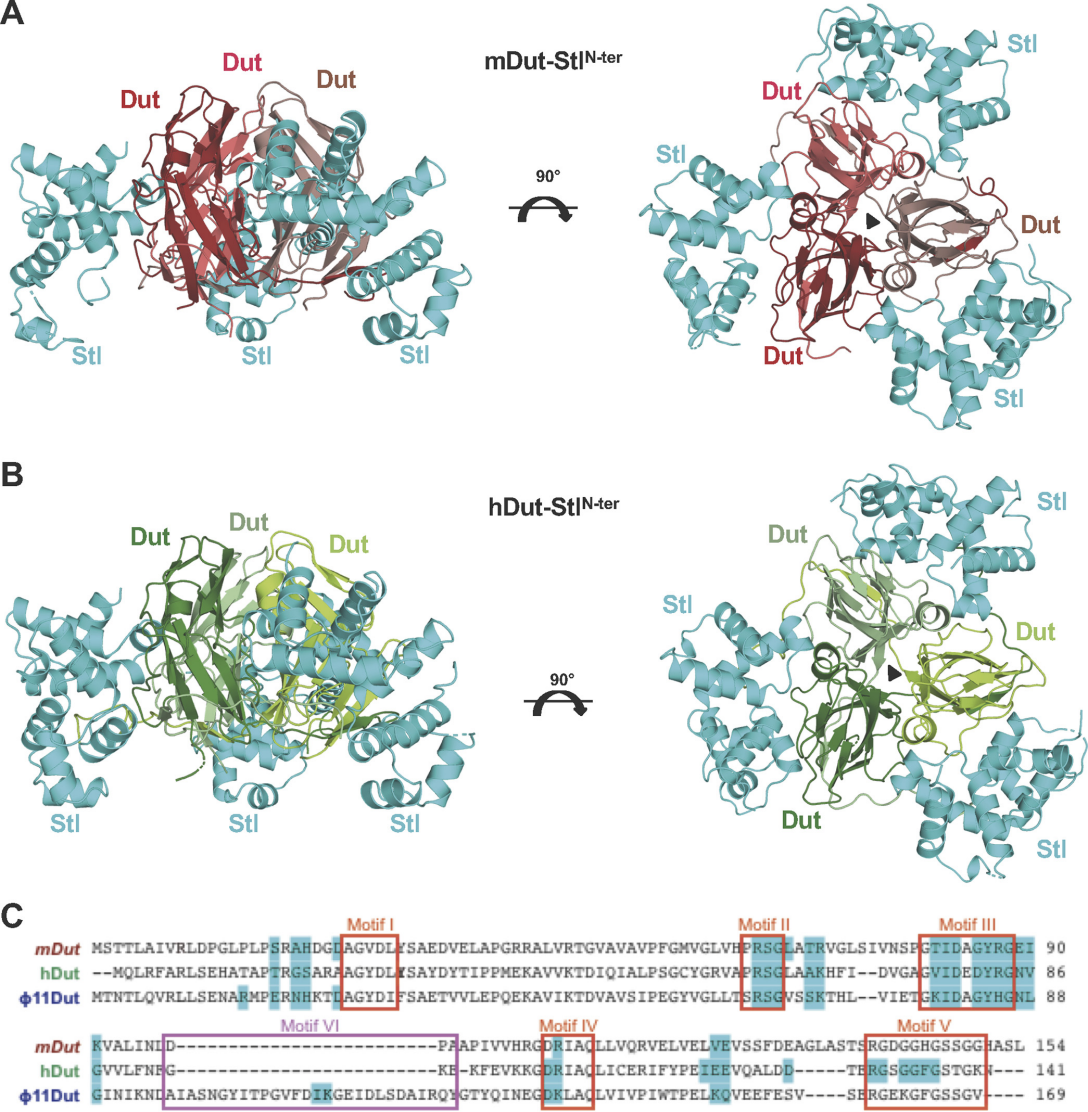
Crystal structures of mDut-Stl^N-ter^ and hDut-Stl^N-ter^ complexes. (A and B) Cartoon representation of Stl^N-ter^ in complex with Duts from M. tuberculosis (A) and human (B). For both complexes, two orthogonal views are shown. Representation scheme: (A) each protomer of trimeric mDut is colored in red and Stl^N-ter^ protomers are in cyan; (B) each protomer of trimeric hDut is colored in green and Stl^N-ter^ protomers are in cyan. (C) Alignment of mDut, hDut, and ϕ11 Dut. The residues of each Dut which interact with Stl^N-ter^ are highlighted in cyan, the catalytic motifs of trimeric Duts are in red boxes, and the S. aureus phage-specific motif VI is in a magenta box.

Once we had confirmed that both prokaryotic and eukaryotic Duts showed high affinity for Stl^N-ter^, the structures of its complex with mDut (mDut-Stl^N-ter^) and with hDut (hDut-Stl^N-ter^) were solved at 2.75- and 1.94-Å resolution, respectively, by molecular replacement using the individual components as searching models (Stl^N-ter^, PDB identifier [ID] 6H49 [11]; mDut, PDB ID 1MQ7 [20]; hDut, PDB ID 1Q5U [21]) (Table 2). The mDut-Stl^N-ter^ complex was crystallized in the space group P2_1_, and the crystal asymmetric unit contains two mDut trimers and six Stl^N-ter^ monomers, forming two mDut-Stl^N-ter^ complexes with a 1:1 stoichiometry in which each mDut trimer interacts with three independent Stl^N-ter^ protomers (Fig. 1A and Fig. S4). The hDut-Stl^N-ter^ complex was crystallized in the space group P2_1_2_1_2_1_, and its asymmetric unit contains one trimer of hDut and three Stl^N-ter^ monomers, with a 1:1 assembly identical to both that which was observed in mDut-Stl^N-ter^ and that which was previously reported for the ϕ11Dut-Stl^N-ter^ complex (11) (Fig. 1B).

**TABLE 2:** Data collection and refinement statistics for mDUT-Stl^N-ter^ and hDUT-Stl^N-ter^ complexes

Parameter	mDUT-Stl^N-ter^^*a*^	hDUT-Stl^N-ter^^*a*^
Data collection		
Beamline	ALBA-XALOC	DLS-I24
Wavelength (Å)	0.979	0.992
Space group	P2_1_	P2_1_2_1_2_1_
Cell dimensions (Å)	a = 79.41, b = 150.43, c = 107.37; α = 90°, β = 98.85°, γ = 90°	a = 77.72, b = 81.80, c = 198.40; α = β = γ = 90°
Resolution (Å)^*a*^	75.21–2.75 (2.85–2.75)	63.11–1.94 (2.01–1.94)
Total no. of reflections	272,538 (19,164)	701,503 (33,067)
No. of unique reflections	61,491 (6,168)	92,354 (7,967)
Completeness (%)	95.04 (95.91)	98.48 (86.22)
Multiplicity	4.40 (4.20)	12.90 (10.00)
Mean *I*/σ (*I*)	8.30 (1.70)	18.90 (2.50)
*R*_pim_	0.05 (0.42)	0.03 (0.24)
CC1/2	0.98 (0.59)	1.00 (0.90)
Refinement		
*R*_work_	0.200 (0.321)	0.215 (0.443)
*R*_free_	0.250 (0.373)	0.255 (0.429)
No. of atoms	12,305	7,045
Protein	12,105	6,585
Water	108	318
Other	16^*b*^	31^*c*^
RMSD, bonds (Å)	0.014	0.015
RMSD, angles (°)	1.86	1.92
MolProbity Clashscore	5.67	5.11
Ramachandran plot		
Preferred (%)	94.70	96.89
Allowed (%)	5.24	2.99
Outliers (%)	0.06	0.12
PDB code	7PWX	7PWJ

a Numbers in parentheses indicate values for the highest-resolution cell.

b Atoms correspond to 8 ethylene glycol, 4 polyethylene glycol, and 4 Tris molecules.

c Atoms correspond to 19 ethylene glycol, 6 sulfate, and 6 glycerol molecules.

The structures of the complexes of mDut and hDut with Stl^N-ter^ confirm not only the 1:1 binding stoichiometry proposed by the biochemical assays (see above [11]) but also the mechanism of trimeric Dut recognition by Stl observed in the complex of Stl^N-ter^ with ϕ11Dut, its natural target (11), as well as the mechanism recently described between Stl and Dut from the crustacean Litopenaeus vannamei (lvDut-Stl^N-ter^ [22]). For all these Dut-Stl^N-ter^ complexes, the Dut maintains its trimeric state and binds three independent Stl monomers, each of which uses predominantly its middle domain to interact with individual active centers of trimeric Dut.

In the mDut-Stl^N-ter^ structure, the two independent complexes are largely identical with a root mean square deviation (RMSD) of 1.1 Å for the superimposition of 642 Cα atoms (Fig. S4) corresponding to the Dut trimer and the three Stl^N-ter^ protomers. In both complexes, clear and traceable electron density is present for the entire Dut protomers, except for the eight C-terminal residues corresponding to the catalytic P-loop motif V, which covers the active center of the enzyme once the nucleotide has been bound. In the complex, the Dut active center is occupied by the Stl^N-ter^ molecule, forcing the projection of this P-loop into the solvent. On the other hand, for all the Stl molecules, the eight N-terminal and the three C-terminal residues are not visible, supporting their nonparticipation in the interactions with the Dut that allows them a high flexibility. In addition, two of the Stl protomers, one for each of the complexes, show regions of the N-terminal DBD (residues 33 to 53 and 59 to 62 in one protomer and residues 38 to 39 and 47 to 61 in the other) where electron density is so weak that it prevents the structure from being traced (Fig. S5), indicating that this domain of the repressor has a greater freedom of movement in the complex due to its weak participation in the interaction with the Dut.

In the hDut-Stl^N-ter^ complex, the three Stl protomers are well defined with the exception of the 8 to 10 N-terminal residues for which, as in the case of the mDut-Stl^N-ter^ complex, no electron density is observable. Surprisingly, in the case of the hDut, the structure allows not only the trace of the main body of each Dut protomer but also the C-terminal motif V (Fig. 1B and Fig. S1). This P-loop, unable to position over the active center occupied by the repressor, is projected toward the Stl and introduces its C-terminal end between the middle domain and the DBD of Stl. This structure gives molecular insight into the contribution of this catalytic motif in Stl binding, which was previously proposed by biochemical and genetic data on the interaction of Duts from S. aureus phages with Stl (7, 11, 16) and recently observed in the structure of the lvDut-Stl^N-ter^ complex (22).

### Dut trimer is the target of Stl.

**FIG 2:**
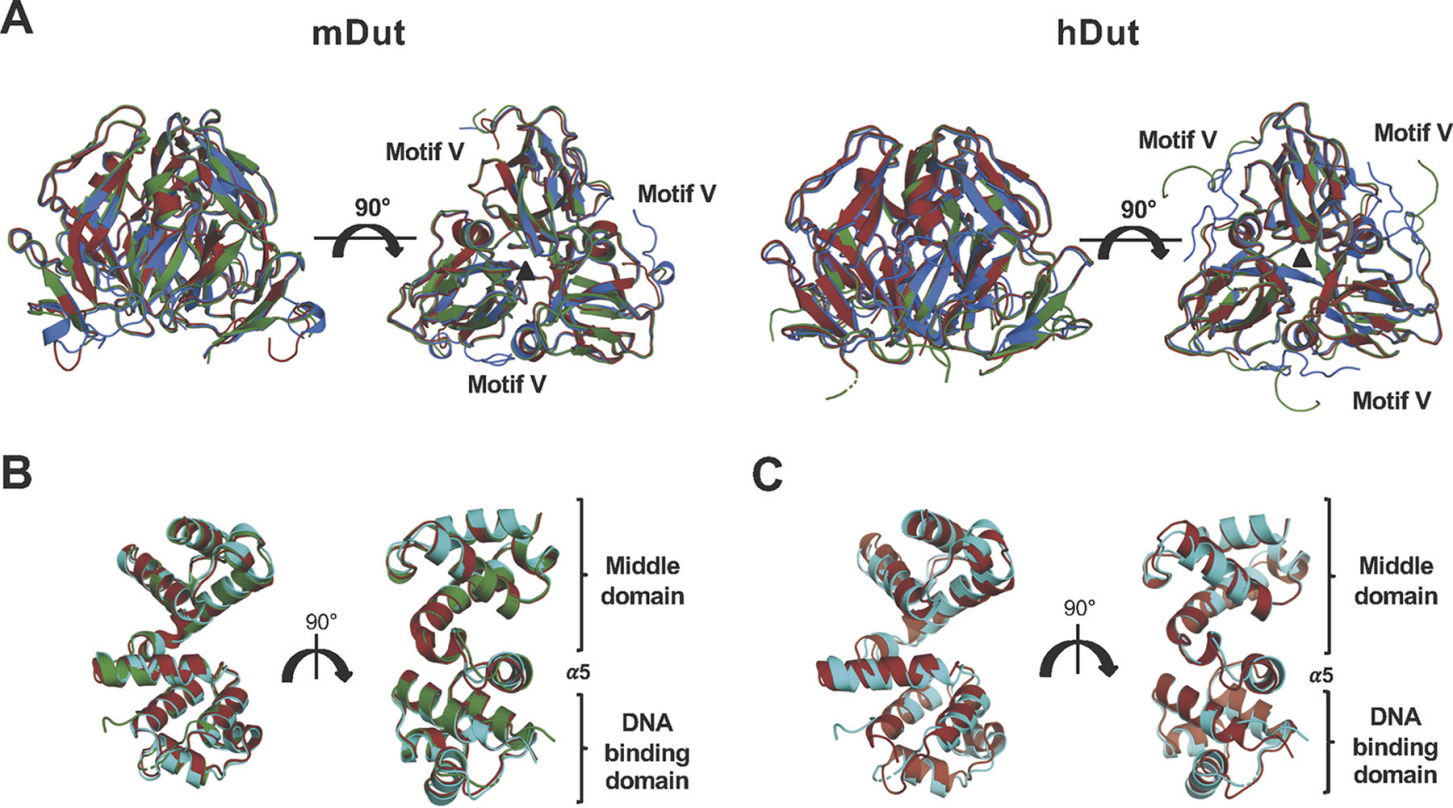
Trimeric Duts present a competent conformation for Stl binding. (A) Superimposed cartoon structures of mDut (left) and hDut (right) in their apo (red), dUTP-bound (blue), and Stl^N-ter^-bound (green) states. Motif V is disordered in the apo state whereas it is ordered over the active center in the nucleotide-bound state. (B) Superimposed cartoon structures of Stl^N-ter^ bound to mDut, hDut, and ϕ11 Dut (red, green, and cyan, respectively). (C) Superimposed cartoon structures of Stl^N-ter^ free and bound to mDut (red and cyan, respectively). Two orthogonal views are shown in all the panels.

The binding of Stl^N-ter^ to ϕ11Dut showed negligible structural changes in the Dut trimer (11). To test whether prokaryotic and eukaryotic Dut trimers are also the conformational targets, we compared the structures of these Duts in complex with Stl^N-ter^ with those in their corresponding free forms (apo and nucleotide bound). The six mDut monomers from the two mDut-Stl^N-ter^ complexes in our structure showed an RMSD below 0.9 Å compared with the free mDut monomers from both the apo (PDB ID 1MQ7) and dUTP-bound (PDB ID 1SJN) forms (Fig. 2A). Similar results were observed with hDut and the hDut-Stl^N-ter^ complex, which showed RMSDs lower than 0.6 Å compared to hDut in apo (PDB ID 1Q5U) or dUDP-bound (PDB ID 1Q5H) forms (Fig. 2A). For the latter comparison, the C-terminal motif V was not included since, although it is visible in both structures, the motifs are projected in different directions due to the presence of Stl or dUDP. Not only do the individual protomers in the complexes possess identical conformations, the trimers in their free and Stl-bound forms also have the same conformation (Fig. 2A). The structural comparison of mDut trimers in apo and dUTP-bound forms (PDB IDs 1MQ7 and 1SJN, respectively) with the Stl^N-ter^ complex showed RMSDs as low as 0.6 Å and, in the case of the hDut trimer, RMSDs lower than 0.5 Å with respect to its apo or dUDP-bound forms (PDB IDs 1Q5U and 1Q5H, respectively), supporting that the target of Stl is the Dut trimer.

Interestingly, a detailed view of this structural comparison shows that the trimer of mDut in complex with Stl is more similar to the nucleotide-bound form than the apo form. The differences are mainly concentrated in the unique and short α helix (mDut residues 65 to 70) from the catalytic motif III (Fig. S1). In the Apo mDut, this helix is distorted, losing its helical topology, whereas in the nucleotide- and Stl-bound forms it folds as identical α helices (Fig. S6). This observation indicates that Stl recognizes the Dut active center in its competent conformation for nucleotide binding, in agreement with the capacity of Stl to inhibit the enzymatic activity of Dut (6, 14).

Similarly, we analyzed the conformational changes induced in Stl by the recognition and binding to the different trimeric Duts. With the exception of two Stl monomers in the mDut-Stl^N-ter^ complex, which presented a greater flexibility in their DBDs that prevented the tracing of some areas (Fig. S5), the remaining Stl protomers showed an almost identical conformation regardless of whether they were bound to a phage, bacterial, or human Dut (Fig. 2B), yielding RMSDs between 0.3 and 1.1 Å (superimposition of 142 to 146 residues). In some cases, the differences were greater between Stl protomers within the same complex than between complexes from different species (Fig. S5). Marginally larger differences were observed when comparing Stls in complex with the Stl free form (PDB ID 6H49) (Fig. 2C), although in any case these differences exceeded RMSDs of 1.6 Å, indicating that the N-terminal portion of the Stl protomer has evolved to acquire a conformation competent to recognize the active center of trimeric Duts, which is highly conserved from phages to human.

### Stl mimics the nucleotide to interact with trimeric Duts.

**FIG 3:**
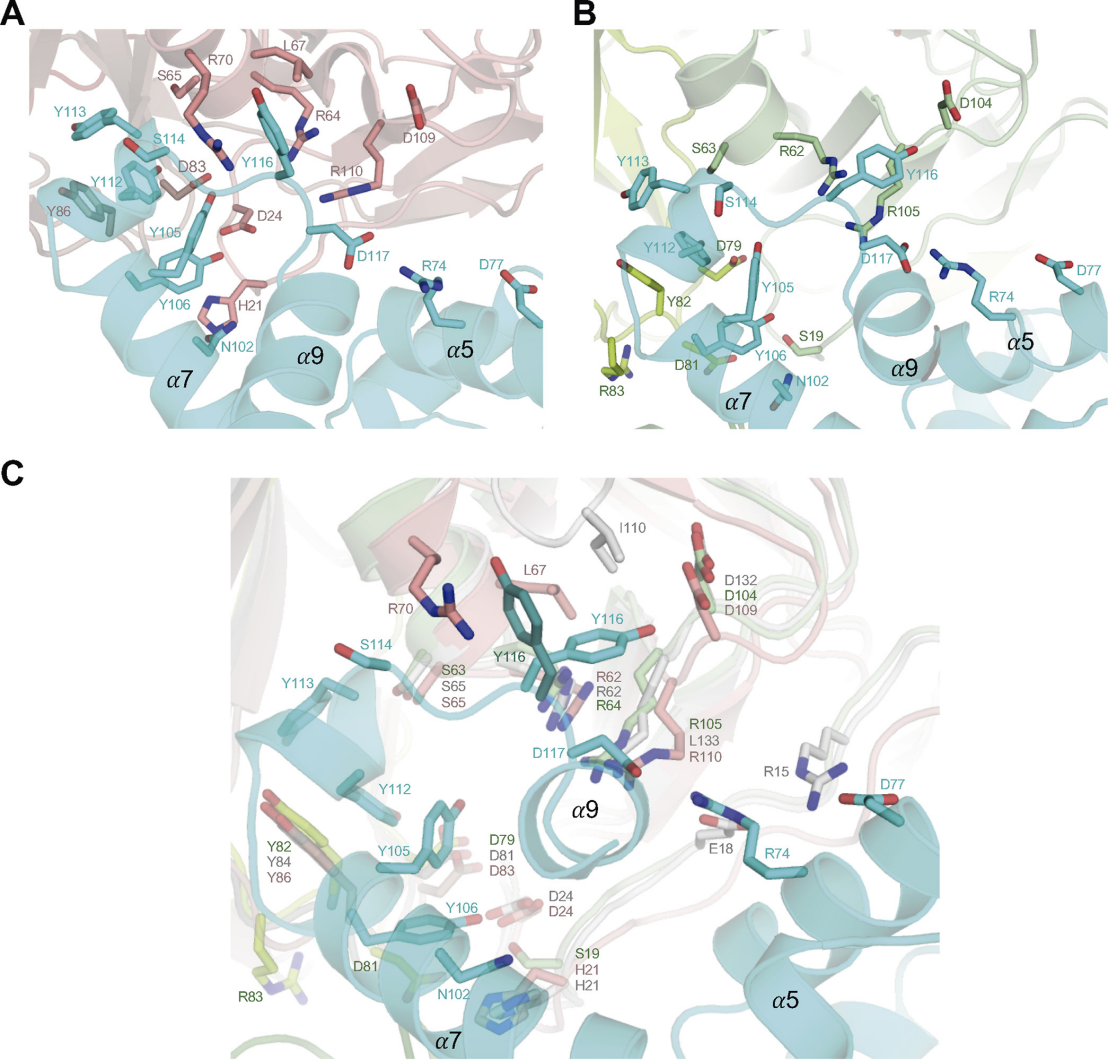
Stl mimics substrate interactions. (A and B) Close-up view of an active center of the trimeric mDut (A) and hDut (B) in complex with Stl^N-ter^ monomer. The main chain is represented in the cartoon, and mDut monomers are colored in red (A), hDut in green (B), and Stl^N-ter^ monomer in cyan. The residues involved in Dut-Stl interaction are shown in stick representation, labeled and colored by atom type with the carbons in the same color as the corresponding protomer. (C) Close-up view of the superimposed active centers.

The analysis of the structures of mDut-Stl^N-ter^ and the hDut-Stl^N-ter^ complexes shows that Stl is using a similar strategy to interact with prokaryotic and eukaryotic trimeric Duts. In both complexes, the repressor inserts the middle domain, specifically the helix α8 and its linkers with helices α7 and α9, into the catalytic site of Dut, in the same way that has been shown previously in ϕ11Dut-Stl^N-ter^ (Fig. 3) (11). From these Stl structural elements, the residues Y112 and Y113 interact with the conserved Asp and Tyr residues of trimeric Dut motif III (mDut D83 and Y86, hDut D79 and Y82, ϕ11Dut D81 and Y84), taking up the place of the substrate ribose, pyrimidine ring, and catalytic water (Fig. 3 and Fig. S7). Meanwhile, Y113 together with Y105 and Y116 also mimics the nucleotide by interacting with conserved Dut catalytic residues, which mediate contacts by interacting with the phosphates of the nucleotide such as the Arg and Ser of motif II (mDut R64 and S65, hDut R62 and S63, ϕ11Dut R64 and S65) and the Tyr of motif III (mDut Y86, hDut Y82, and ϕ11Dut Y84) (Fig. 1C, Fig. 3, Fig. S1 and S7, and Tables S2 and S3). The nucleotide mimicry mechanism of binding carried out by the Stl helix α8 represents the main anchor point to the Dut, which is reinforced with additional interactions that recognize partially conserved residues in Duts. This is the case for Stl Y106, which interacts with conserved or partially conserved residues from motifs I and III, or Stl S114 and D117, which interact with positively charged residues (Arg or Lys) located in Dut motifs II and IV (Fig. 3, Fig. S1 and S7, and Tables S2 and S3). Our previous work with S. aureus phage Duts has shown that the presence of a basic residue of Arg or Lys in motif IV implied changes in affinity for Stl (16). Given the variability in this residue among the solved complexes (mDut R110, hDut R105, and ϕ11Dut K133), we looked in greater detail at the structures. In all the structures, the basic residue is salt bridged with Stl D117 and makes hydrophobic and polar interactions with Y116, but this second interaction differs depending on the Dut recognized by Stl. In the original ϕ11Dut-Stl^N-ter^ complex, Y116 is completely inserted into the Dut core and interacts with residues from catalytic motifs II (R64) and IV (K133) and the phage-specific motif VI (I110) (11). The Y116 side chain conformation in the hDut-Stl^N-ter^ is identical to that in the ϕ11Dut complex, although its placement is not restricted, because this Dut lacks motif VI. In contrast, the absence of motif VI is exploited in the complex with mDut where Y116 presents, in the different Stl subunits, rotameric conformations alternative to that observed in the ϕ11Dut and hDut complexes. As in phage and human complexes, Y116 from some Stl protomers interacts with mDut D109 and R110, but in other protomers, the rotamer is identical to that observed in the Stl free form, losing these interactions and forming new ones with L67 and R70 in motif II (Fig. 3C). Intermediate conformations between the two rotamers are also observed in some protomers. While these changes confirm the versatility of Stl in mimicking nucleotide interactions, they could also explain the Stl affinity differences shown for the different Duts. Moreover, differences in affinity could also be explained by peripheral interactions provided by the Stl residues in the DBD and helix α5, which vary according to the recognized Dut (Tables S2 and S3).

### Motif V is an active element in Stl binding.

**FIG 4:**
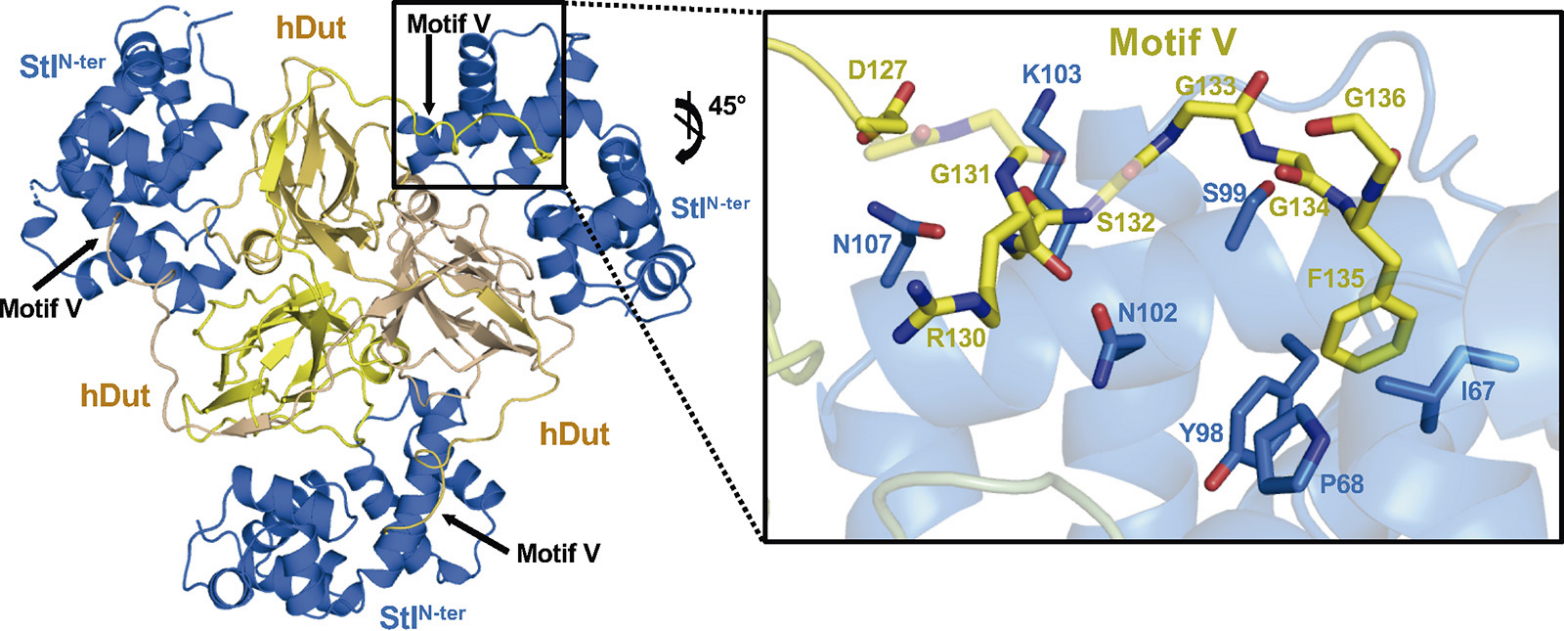
Motif V of hDut is involved in Stl recognition and binding. (Left) Cartoon representation of trimeric hDut (yellow tones) bound to three Stl^N-ter^ protomers (navy blue) shown as the Dut motifs V are inserted in Stl^N-ter^ to ensure the complex formation. (Right) Close-up view of the interaction between hDut motif V and Stl^N-ter^ with the residues participating shown as sticks and labeled.

The participation of motif V of trimeric Duts in the recognition and binding to Stl has been controversial (7, 16, 23). Our previous work with trimeric Duts from S. aureus phages showed that the level of participation of motif V varies between Duts (16). The recently reported lvDut-Stl^N-ter^ complex shows the first example of how the Dut C-terminal end would recognize Stl (22). In the two lvDut-Stl^N-ter^ complexes present in the crystal structure, the C-terminal tail is visible in 4 of the 6 Dut subunits, projected from the Dut core to reach the Stl and introducing a Phe residue from motif V into a hydrophobic pocket located between the DBD and middle Stl domain. In the hDut-Stl^N-ter^ complex, all the Dut C-terminal tails are well ordered, showing a similar mechanism of Stl recognition and complex stabilization by introducing a conserved Phe from Dut motif V in the Stl interdomain hydrophobic pocket (Fig. 4 and Fig. S8). However, C-terminal Dut tail binding is a consequence of the interaction of more than one residue, since the Stl repressor exploits residues from loop α4-α5 (G66, I67, and P68) and the helix α7 (Y98, S99, N102, K103, and N107) to interact with the main and side chains of different Dut motif V residues (D127, R130, G133, G134, and G136) (Fig. 4 and Table S3). On binding to Stl, the Dut C-terminal tail shows a totally different placement from that observed for nucleotide recognition. In order to allow this drastic change of the C-terminal tail direction, the flexibility provided by several Gly residues and the pivoting interactions of Arg130 at the beginning of the tail are fundamental. Therefore, Stl recognition and binding are brought about by multiple conserved residues from motif V in different ways, anchoring the C-terminal tail by the Phe, providing flexibility by the Gly residues, and being a hinge through the Arg residue (Fig. 4).

In contrast, no density attributable to the C-terminal tail was found in the mDut-Stl^N-ter^ complex. The main difference between hDut and mDut in this region corresponds to the replacement of the anchoring Phe by a His (H135), a polar residue whose accommodation in the Stl hydrophobic pocket is unfavorable energetically. Therefore, we hypothesized that the Phe-His change eliminated the main anchor point of the C-terminal tail in Stl, preventing the participation of this structural element in receptor binding. In order to confirm our proposal, we generated an mDut mutant where H135 was replaced by a Phe (mDut^H135F^) and analyzed its binding to Stl. It is worth noting that this position in motif V is occupied by an aromatic residue, typically Phe, which stacks over the uracil ring, and that previous replacement of the mDut His135 by another aromatic residue (Trp) had minimal effect on the dUTPase activity (18). Using the BLI technique, a *K_D_* of 12.45 nM was calculated for the mDut^H145F^ binding to Stl^N-ter^, representing a 3-fold improvement in affinity relative to wild-type mDut (Table 1 and Fig. S3). This gain-of-function supports the participation of motif V in the binding once the anchoring Phe is introduced. Therefore, these results put an end to the controversy about the involvement of the C-terminal Dut domain (motif V) in the recognition and binding to Stl, highlighting the conserved Phe in motif V as a key actor in this process.

### Stl is a universal Dut binder.

**FIG 5:**
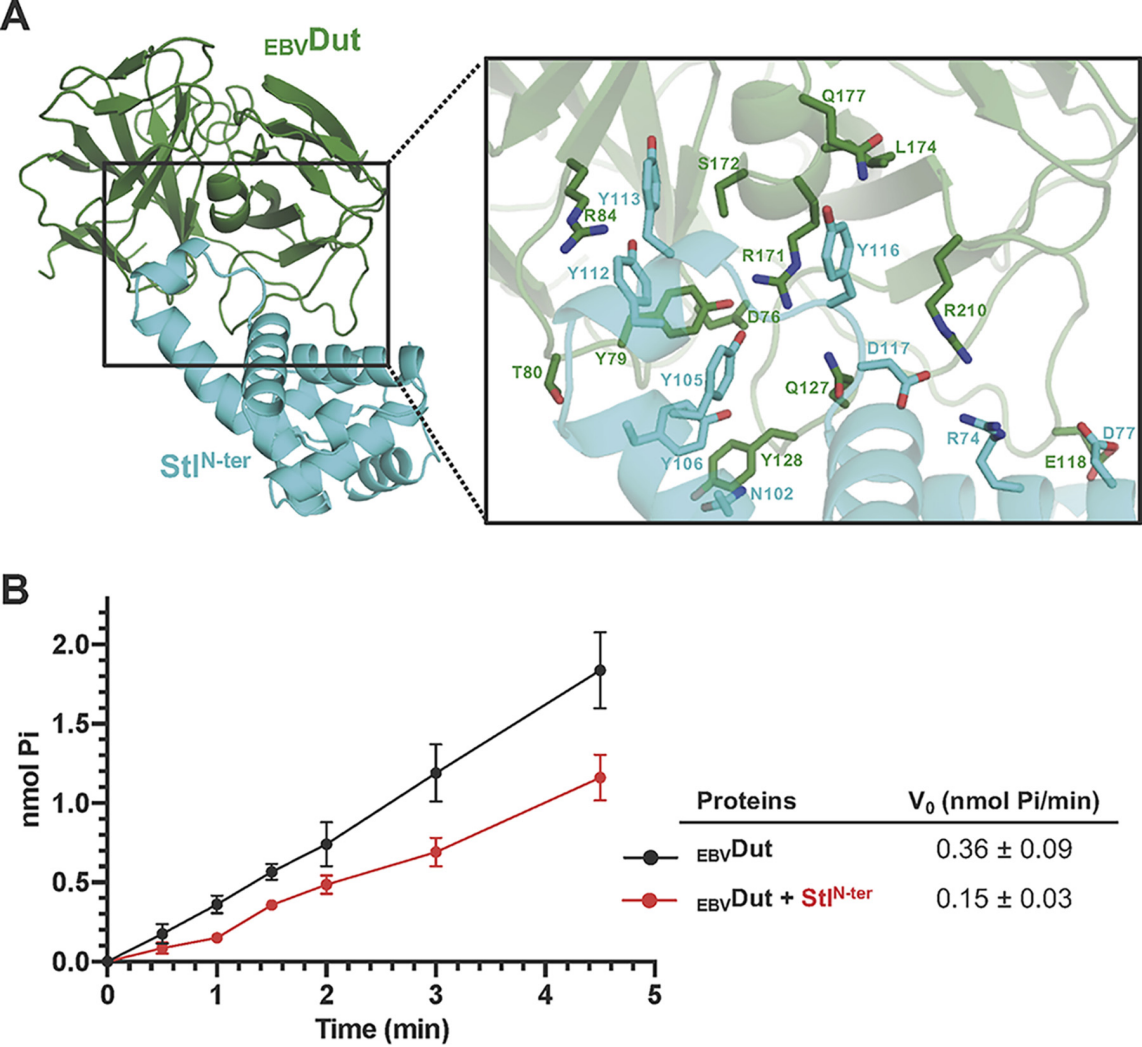
Stl recognition of monomeric Duts. (A) Model of monomeric Dut-Stl^N-ter^ complex. (Left) Cartoon representation of the _EBV_Dut-Stl^N-ter^ model with Dut and Stl colored in green and cyan, respectively. (Right) Close-up view of the monomeric Dut active center where the Stl is accommodated, showing in stick form the residues involved in Dut-Stl interactions. (B) Stl inhibits _EBV_Dut activity. The enzymatic activity of _EBV_Dut as production of P_i_ was calculated in the absence (black) or presence (red) of Stl^N-ter^. The graph shows the results and standard deviations from three independent assays.

Monomeric Duts originated from trimeric Duts by gene duplication and in tandem fusion, and because of that, both families exhibit highly conserved active centers and catalytic mechanisms (24, 25). We therefore considered whether Stl would also recognize monomeric Duts, thus being a universal binder of Duts. For a deeper insight into this hypothesis, we followed an *in silico* approach to produce a model of an Stl^N-ter^-monomeric Dut complex by taking advantage of the structural information available in the PDB and that provided here. Since Stl mimics the nucleotide to interact with trimeric Duts, we exploited the conservation in the catalytic mechanism between monomeric and trimeric Duts to generate the model by superimposing the nucleotides present in the active centers of both Dut types. We selected the monomeric Dut from Epstein-Barr virus (_EBV_Dut; PDB ID 2BT1 [25]), and the trimeric Duts from M. tuberculosis (PDB ID 1SIX [20]) and humans (PDB ID 2HQU [26]), since their complexes with Stl are presented in this work and are representative of prokaryotic and eukaryotic Duts, respectively. Once the active centers of monomeric and trimeric Duts were aligned by superimposing their nucleotides, confirming their structural relationship (Fig. S9), Stl was positioned on the monomeric _EBV_Dut by superimposing the Dut component of the corresponding trimeric Dut-Stl^N-ter^ complexes. Regardless of the trimeric Dut used (mDut or hDut) to position Stl on the monomeric Dut, the results obtained were similar and no steric problems were observed in any of the complexes (Fig. 5A). The models show that Stl inserts its α8 helix into the _EBV_Dut active center and that Stl could also exploit its Tyr (Y105, Y112, Y113, and Y116) residues to recognize conserved catalytic residues of the monomeric Dut, highlighting the interactions of Y112 and Y113 with the catalytic Asp (D76) and the uracil ring recognition Tyr (Y73) residues of _EBV_Dut (Fig. 5A and Table S4). Likewise, the _EBV_Dut-Stl^N-ter^ models show that other Stl interactions with conserved residues of motifs II and IV in trimeric Duts are also replicated for monomeric Dut (Table S5). In this way, Stl D117 mediates a salt bridge with _EBV_Dut R280 equivalent to that observed with Lys/Arg from motif IV in trimeric Duts, and _EBV_Dut R171 and S172 also replicate the interactions with Stl provided for identical residues (mDut R64/S65 and hDut R62/S63) in trimeric Dut motif II. Although some conserved Stl-trimeric Dut interactions are not replicated in the _EBV_Dut-Stl^N-ter^ complex (e.g., the Stl S114 interaction with a positively charged residue in trimeric motif II), the majority of interactions are replicated (Table S5), supporting that Stl can bind and inhibit monomeric Dut. Indeed, the Stl positioning is appropriate for the _EBV_Dut C-terminal P-loop, which is highly flexible as deduced from its lack of density in the PDB available structures, to recognize Stl in a manner similar to that observed in the hDut-Stl^N-ter^ complex. To experimentally check this inhibitory capacity, we produced and purified _EBV_Dut and analyzed its dUTPase activity in the presence and absence of Stl^N-ter^. The enzymatic assays showed a decrease in dUTPase activity of about 60% in the presence of Stl^N-ter^ (Fig. 5B). A comparable inhibition (40 to 80%) of dUTPase activity induced by Stl on mDut, hDut, or D. melanogaster Dut was previously reported (13–15), supporting a monomeric-Stl interaction similar to that observed with these trimeric Duts.

**FIG 6:**
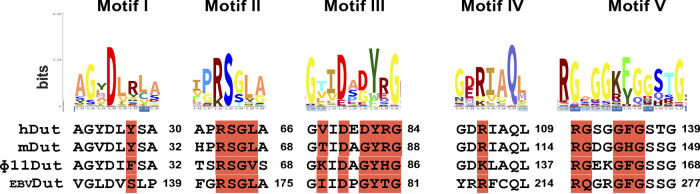
Sequence analysis of trimeric and monomeric Dut regions involved in Stl^N-ter^ recognition and binding. In the upper part, the logos of the five Dut catalytic motifs are shown. The height of the amino acid symbol is proportional to its frequency at the specific position. In the lower part, the sequences for hDut, mDut, ϕ11Dut, and _EBV_Dut are aligned with the logo, and the positions participating in interactions with Stl (crystal structures and model for trimeric and monomeric Duts, respectively) are highlighted with a red background.

Finally, in order to evaluate the possible universality of Stl as a Dut binder, we performed a sequence analysis of trimeric and monomeric Duts, which showed in most cases that the positions in the Dut motifs mediating interactions with Stl are highly conserved, with the exception of motif I, whose contribution to binding is minimal (Fig. 6). It should be noted that the inclusion of monomeric Duts, which have a reshuffled sequence relative to the trimeric Duts (25), means that only a part of the catalytic motifs can be correctly aligned, decreasing the degree of conservation. The conservation is especially high in residues from motifs II and III, with more variation in motif IV residues, which, as our previous *in vitro* and *in vivo* results have shown (7, 11, 16), could modulate the affinity between Duts and Stl. This high conservation supports that Stl can recognize and interact with a wide range of trimeric and monomeric Duts, proposing this protein as a universal Dut binder. Given the inhibitory nature of this interaction, the results shown here support the proposed use of Stl as a proteinaceous inhibitor of Duts (15), which could be shared by Stls from other SaPIs that have high sequence homology in their middle and N-terminal domains (6).

## DISCUSSION

We had previously demonstrated that different phage dUTPases, which are structurally unrelated, are able to interact with the SaPIbov1 Stl repressor and had deciphered the particularities of the molecular mechanism of recognition for each of them (6, 9, 11). However, this capacity is not limited to Duts from phages, since it has been shown that other trimeric Duts from distant organisms in the “tree of life” are also able to interact with Stl (13–15). Given the exceptional capacity of Stl to interact with proteins that maintain identical enzymatic activity only but differ in their structure, we wondered if the molecular mechanism used to recognize trimeric Duts from prokaryotes and eukaryotes would be similar to that used to recognize Duts from phages.

The complexes of Stl with the mycobacterial and human Duts show that Stl recognizes and binds to the Duts by exploiting interactions with almost all the conserved catalytic residues of these enzymes, and thus, Stl is a universal Dut repressor in multiple organisms along different directions of the tree of life. The human-Stl complex confirms our previously proposed and recently observed implication of the C-terminal motif V in trimeric Dut binding. Contradictory results derived from analyses performed with different Duts from different S. aureus phages, 80α and ϕ11, led to disagreements about the involvement of motif V in the recognition and binding to Stl. The structural data presented here and those recently described for the Stl in complex with Dut from L. vannamei (22) confirm and provide visualization of our previously proposed model. For this purpose, Stl exploits interactions with one of the most conserved residues of trimeric Dut motif V, a phenylalanine that is crucial in the catalytic mechanism by positioning itself over the uracil ring once the nucleotide is bound, stabilizing the P-loop. This phenylalanine is inserted into a hydrophobic pocket of Stl generated by the interface between its N-terminal DNA-binding domain and the middle domain, which recognizes the Dut nucleotide binding pocket. Although this Phe is highly conserved among trimeric Duts, which strongly supports Stl as a possible universal inhibitor of these enzymes, some variations in this position have been observed, as is exemplified by the Dut of M. tuberculosis. In this case that residue is a histidine, which could penalize its affinity for Stl, since its positioning in the hydrophobic pocket is energetically unfavorable. We confirmed this hypothesis by changing the His of M. tuberculosis Dut in this position to a Phe, and it showed that the mutant Dut has 3-times-higher affinity for Stl than the wild-type Dut. This result definitively confirms the participation of motif V in the binding of trimeric Dut to Stl and is further evidence of the high level of selection that Stl has followed to recognize most of the catalytic residues of these Duts in such a way as to prevent the generation of Dut escape mutants while retaining enzyme activity. Therefore, this sophisticated mechanism of Stl confirms the evolutionarily favorable strategy of binding by mimicking the target substrate and not by the recognition of a specific domain or sequence, which guarantees the success in SaPI derepression and dissemination.

At this point, we contemplated another relevant aspect. Of the three families of Duts (monomeric, dimeric, and trimeric), the binding strategy of Stl had been proven for both dimeric and trimeric forms (11), and given that trimeric and monomeric Dut catalytic motifs are highly conserved, we considered the possible spreading of the mimicry strategy of Stl to monomeric Duts. These Duts are specific to herpesviruses, which have only vertebrate hosts; therefore, Stl could not have been evolutionarily selected to recognize these Duts, as happened with the trimeric and dimeric forms. However, the structural and mechanistic relationship between trimeric and monomeric Duts (25), as well as the versatility shown by Stl in binding structurally unrelated proteins, opened up the possibility for Stl-monomeric Dut interaction. Our *in silico* study generating a structural model of the Stl-monomeric Dut complex, together with deep sequence analysis, supported the versatility shown by this repressor. Our model shows no steric hindrance in the binding of Stl to monomeric Duts and a similar mimicry mechanism, exploiting interactions with catalytic residues in the Dut active center. Four Stl tyrosines (Y105, Y112, Y113, and Y116) mimic the dUTP substrate interacting with conserved residues in monomeric Duts. Indeed, analysis of more than 10,500 sequences of Duts revealed that catalytic residues from four of the five motifs (Arg and Ser from motif II; Val, Ile, Asp, Tyr, and Arg from motif III; Asp and Arg from motif IV; Ile, Gly, and Phe from motif V) are highly conserved in both monomeric and trimeric Duts. Finally, we experimentally confirmed that the monomeric Dut for Epstein-Barr virus is enzymatically inhibited by Stl in a manner similar to that of other prokaryotic and eukaryotic trimeric Duts, supporting the universality of Stl as a direct competitor of dUTP and a natural inhibitor of Duts.

## MATERIALS AND METHODS

### Gene cloning.

General DNA manipulations were performed using standard procedures. The gene encoding dUTPase from M. tuberculosis was amplified from genomic DNA (strain H37Rv) using the primers mDut-Fw and mDut-Rv (see Table S1 in the supplemental material). The PCR product was cloned in pETNKI 1.1 vector (NKI Protein Facility LIC vector system) previously digested with KpnI (Fermentas) and treated with T4 DNA polymerase (New England Biolabs [NEB]). The mutant mDut H145F was designed and made using the Q5 site-directed mutagenesis kit (NEB) and the primers mDutH145A-Fw and mDutH145F-Rv (Table S1). The synthetic gene encoding the common region of nuclear and mitochondrial isoforms of the human dUTPase (UniProtKB access code H0YNW5, Fig. S10) with the overhangs hDut-FW and hDut-RV was manufactured by the IDT Company and was subsequently cloned into a pET28a vector using the NEBuilder HiFi DNA assembly cloning kit (NEB) and the primers pET28A-FW and pET28a-RV (Table S1).

### Expression and protein production.

mDut and its mutant mDut^H145F^ were produced in Escherichia coli BL21(DE3) in LB medium supplemented with kanamycin at 33 μg/mL. hDut was produced in E. coli Rosetta in LB medium supplemented with kanamycin and chloramphenicol, both at 33 μg/mL. Cultures were grown at 37°C up to an optical density at 600 nm (OD_600_) of ~0.5 when the protein expression was induced with 0.5 mM isopropyl-β-d-thiogalactopyranoside (IPTG) for 16 h at 20°C. After induction, cells were collected by centrifugation at 4,000 × *g* for 30 min at 4°C. The cellular pellet was washed with 1× phosphate-buffered saline (PBS), resuspended in buffer A (0.2 M Tris-HCl, pH 8, 0.4 M NaCl for mDut or 0.1 M Tris-HCl, pH 8, 0.3 M NaCl for hDut), and sonicated after adding 1 mM phenylmethylsulfonyl fluoride (PMSF). Lysates were centrifuged at 15,000 × *g* for 35 min at 4°C, and the soluble fraction was loaded onto a preequilibrated His-Trap HP column (GE Healthcare) with the respective buffer A for affinity chromatography purification. The columns were washed with 10 column volumes (CV) of buffer A supplemented with 10 mM imidazole, and proteins were eluted with buffer B (0.15 M Tris-HCl, pH 8, 0.4 M NaCl, 0.25 M imidazole for mDut and mDut^H145F^ and 0.04 M Tris-HCl, pH 8, 0.2 M NaCl, 0.25 M imidazole for hDut).

When required, His tag from purified Duts was removed by digestion using glutathione *S*-transferase (GST)-tagged PreScission protease at a molar ratio of 1:50 (protease/Dut) at 4°C for 16 h and then loaded onto tandem His-Trap and GST-Trap columns to remove the undigested proteins and the GST-protease from the sample. Purified Duts were concentrated through an Amicon Ultra system (30-kDa cutoff) and further purified by size exclusion chromatography. The proteins were loaded onto a preequilibrated Superdex S75 column (GE Healthcare) with buffer C (0.1 M Tris-HCl, pH 8, 0.4 M NaCl for mDut and mDut^H145F^ and 0.02 M Tris, pH 8, 0.125 M NaCl, 1 mM MgCl_2_ for hDut). After an isocratic elution, fractions were analyzed by SDS-PAGE, and those with the highest purity were concentrated, flash frozen in liquid nitrogen, and stored at −80°C.

The proteins ϕ11Dut and Stl^N-ter^ (residues 1 to 156) were expressed and purified as previously described (11).

### Native PAGE.

A fixed concentration of Stl^N-ter^ (11 μM) was mixed with increasing concentrations of Dut from a molar ratio of 0.5:1 to that of 4:1 (Dut/Stl^N-ter^) in buffer containing 75 mM HEPES, pH 7.5, 250 mM NaCl, and 5 mM MgCl_2_ and incubated overnight at 4°C. Samples were loaded in 8% polyacrylamide native gels. Gels were prerun in 25 mM Tris-HCl, pH 8.3, 1.44% (wt/vol) glycine buffer, at 4°C, 150 V for 1 h. Soon after, samples were loaded and the electrophoresis was performed in the same buffer and at the same temperature and voltage for 135 min for ϕ11Dut and 210 min for hDut and mDut.

### Biolayer interferometry assays.

Binding studies were performed on the Octet system (Sartorius). All kinetic assays were performed in freshly prepared and filtered buffer containing 75 mM HEPES (pH 7.5), 250 mM NaCl, 5 mM MgCl_2_, 1% bovine serum albumin (BSA), 0.005% Tween 20, and 10 mM imidazole at 28°C. The samples were dispensed in dark 96-well polypropylene plates and placed on the shaker at 1,000 rpm. Sequentially, His-tagged Duts (at 218.7 nM) were captured on nickel-nitrilotriacetic acid (Ni-NTA) biosensors for 280 s followed by one step of 200 s in buffer to remove Dut excess and 60 s to establish the baseline. For the association, the Dut captured was exposed to decreasing concentrations of untagged Stl^N-ter^, from 218.7 nM to 3.4 nM in serial one-half dilutions, for 120 s. Dissociation was performed in buffer for 120 s. Data were analyzed using the Octet Data Analysis HT program (Sartorius) and fitted to a 1:1 kinetic model.

### dUTPase activity assay.

A Malachite Green phosphate assay was used to analyze the inhibitory capacity of Stl^N-ter^ on the dUTPase activity by quantifying P_i_ released (27, 28). Assays were carried out in 50 μL of reaction buffer containing 20 mM HEPES (pH 7.5), 250 mM NaCl, 10 mM MgCl_2_, 10 mM imidazole, and 0.0025 U of inorganic pyrophosphatase (Thermo Scientific). The dUTPase activity of 0.3 μg of _EBV_Dut was measured, and 4 μg of Stl^N-ter^ (molar ratio of 1:20, dUTPase/repressor) was included to test the inhibitory activity. In the inhibition assays, Stl and Dut were incubated for 15 min at room temperature before the reaction. The reactions were started by the addition of 15 μM dUTP, samples at different time points (0 to 5 min) were taken, and reactions were stopped by adding 200 μL of acidic Malachite Green solution. After 20 min of incubation at room temperature, the absorbance at 630 nm was measured and the P_i_ production was calculated based on a standard curve of P_i_ included in the assay.

### Crystallization and data collection.

Crystals from both complexes were grown as sitting drops at 21°C with a vapor-diffusion approach. Initial crystallization attempts were set up in the crystallogenesis service of the IBV-CSIC using commercial screening assays JBS I and II (Jena Biosciences) and JCSG+ (Molecular Dimensions) in 96-well plates. Complexes were formed by mixing untagged mDut or His-tagged hDut with untagged Stl^N-ter^, in a 1-to-1 molar ratio (calculated for the monomer), and mixtures were incubated at 4°C overnight prior to the crystallization plate setup. Crystallization drops were generated by mixing equal volumes (0.3 μL) of sample and the corresponding reservoir solution and were equilibrated against 100 μL reservoir solution. The mDut-Stl^N-ter^ complex was crystallized at 17 mg/mL in a reservoir solution consisting of 20% polyethylene glycol (PEG) 10000 and 0.1 M Na-HEPES, pH 7.5. Crystals were cryoprotected with a solution consisting of reservoir solution increased up to 35% PEG 10000 and supplemented with 10% ethylene glycol. The hDut-BovI-Stl^N-ter^ complex was crystallized at 3 mg/mL in a reservoir solution of 1.6 M (NH_4_)_2_SO_4_ and 1 M Li_2_SO_4_, and crystals were cryoprotected with reservoir solution supplemented with 5% glycerol and 10% ethylene glycol.

Diffraction data were collected from single crystals at 100 K on the ALBA (Barcelona, Spain) and DLS (Didcot, UK) synchrotrons. Processing and reduction were done with iMosflm (29) and Aimless (30) programs (CCP4 suite [31]). Data collection statistics are shown in the crystallography table (Table 2).

### Model building.

Crystallographic phases for mDut-Stl^N-ter^ and hDut-Stl^N-ter^ complexes were obtained by molecular replacement using PHASER (32) and the structures of mDut (PDB ID 1MQ7 [20]), hDut (PDB ID 1Q5U [21]), and Stl^N-ter^ (PDB ID 6H49 [11]) as searching models. Final models were generated by iterative cycles of refinement using Refmac (33) and manual rebuilding and optimization with Coot (34). Maximum likelihood was applied in each cycle of restrained refinement, using automatic weighting and experimental sigmas to weight X-ray terms and excluding 5% data for *R*_free_ calculation. The temperature factors were refined as isotropics. TLS refinement was applied in the last cycles of hDut-Stl^N-ter^ complex refinement. Data refinement statistics are given in the crystallography table (Table 2). Atomic coordinates and structure factors have been deposited in the Protein Data Bank with identification codes 7PWX for mDut-BovI-Stl^N-ter^ and 7PWJ for hDut-BovI-Stl^N-ter^.

### Structural analysis.

The CCP4 suite (31) and PISA server (35) were used to analyze interactions in the complexes and to superimpose the structures. The omit map from domain V on the hDut-Stl^N-ter^ complex was calculated using the Phenix suite (36). The residues from domain V (R130 to G136) were the subject of omit selection specifying 3 Å as the solvent exclusion radius.

### Structural modeling of the _EBV_Dut-Stl^N-ter^ complex.

The structural model _EBV_Dut in complex with Stl^N-ter^ was generated by the following steps. As a representative monomeric Dut, we used the structure of _EBV_Dut in complex with the dUTP analogous nucleotide α-β-imino dUTP (PDB ID 2BT1). The Coot program (34) was used to superimpose the nucleotide in this structure with the nucleotides inside one of the active centers of the structures of mDut (PDB ID 1SIX) or hDut (PDB ID 2HQU). The conformation of nucleotides in all the structures is almost identical. In this way, the structure of the monomeric _EBV_Dut was aligned according to its substrate recognition with both trimeric Duts. Then, each one of these trimers of Dut was superimposed with the corresponding trimer of Dut in complex with Stl^N-ter^ (mDut-BovI-Stl^N-ter^ or hDut-BovI-Stl^N-ter^), so that Stl was positioned on the active site of _EBV_Dut with the supposed recognition conformation. At this point, the _EBV_Dut-Stl^N-ter^ models were finished by eliminating the structures of the corresponding trimeric Duts, creating one _EBV_Dut-Stl^N-ter^ complex for the Stl^N-ter^ from mDut-Stl^N-ter^ and another for that from hDut-Stl^N-ter^. Energy minimization of both models was then performed using the Yasara energy minimization server, using the default Yasara force field and minimization values (37). Since the two _EBV_Dut-Stl^N-ter^ models are very similar, the one generated with Yasara with the Stl^N-ter^ from the mDut-Stl^N-ter^ complex structure was used to produce the figures presented in this study.

### Sequence alignment and logo diagram.

In order to determine the amino acid profile and sequence variability of monomeric and trimeric Dut sequences, we performed the following analysis. Dut sequences from the Pfam database under ID PF00692 (14,635 sequences), which include monomeric and trimeric Duts, were downloaded and computationally distributed based on their length. Sequences showed a size mainly between 125 and 225 residues, so those sequences outside this range (generally corresponding to Dut fragments or Dut domains fused to other proteins) were eliminated. The size analysis also showed a bimodal distribution with peaks centered on 150 and 190 residues. Inspection of representative sequences of these peaks indicated that the first one corresponds to trimeric Duts and the second to monomeric Duts plus trimeric Duts carrying insertions (e.g., signaling peptides). Finally, to avoid overrepresentation of certain families, homologs were clustered using a 95% identity coverage with MMseqs2 version 12.113e3 (38). One representative homolog per cluster was selected, resulting in a final data set of 10,616 sequences that were aligned using the MAFFT program version 7.475 (39), and the resulting alignment was visualized using the SeaView program version 5.0.4 (40). The high level of diversity in sequences as a consequence of the large number of proteins included involved empty columns across the alignment. For adding reliability to the resultant logo of the alignment, these empty columns were removed using the “- gappyout” parameter of the trimAl tool version 1.4. rev14 (41), alignment size-dependent guideline. This sequence alignment was uploaded to the Skylign webserver (42) to obtain the corresponding logo, which is a graphical representation of sequence conservation where the frequency of each amino acid residue at the specific position is proportional to the height of its symbol. In the graphic, the presence of a single letter indicates that the residue is invariable, whereas the presence of different letters corresponds to the most common amino acids.

### Data availability.

Atomic coordinates and structure factors have been deposited at the RCSB Protein Data Bank (PDB) (PDB code 7PWX for mDUT-Stl^N-ter^ and 7PWJ for hDUT-Stl^N-ter^).

## References

[1] Humphrey S, San Millán Á, Toll-Riera M, Connolly J, Flor-Duro A, Chen J, Ubeda C, MacLean RC, Penadés JR. 2021. Staphylococcal phages and pathogenicity islands drive plasmid evolution. Nat Commun 12:5845. doi:10.1038/s41467-021-26101-5. 34615859 PMC8494744

[2] Haag AF, Podkowik M, Ibarra-Chávez R, Gallego del Sol F, Ram G, Chen J, Marina A, Novick RP, Penadés JR. 2021. A regulatory cascade controls Staphylococcus aureus pathogenicity island activation. Nat Microbiol 6:1300–1308. doi:10.1038/s41564-021-00956-2. 34518655 PMC7611864

[3] Tormo-Más MÁ, Mir I, Shrestha A, Tallent SM, Campoy S, Lasa Í, Barbé J, Novick RP, Christie GE, Penadés JR. 2010. Moonlighting bacteriophage proteins derepress staphylococcal pathogenicity islands. Nature 465:779–782. doi:10.1038/nature09065. 20473284 PMC3518041

[4] Penadés JR, Christie GE. 2015. The Phage-inducible chromosomal islands: a family of highly evolved molecular parasites. Annu Rev Virol 2:181–201. doi:10.1146/annurev-virology-031413-085446. 26958912

[5] Tormo-Más MA, Ferrer MD, Maiques E, Úbeda C, Selva L, Lasa Í, Calvete JJ, Novick RP, Penadés JR. 2008. *Staphylococcus aureus* pathogenicity island DNA is packaged in particles composed of phage proteins. J Bacteriol 190:2434–2440. doi:10.1128/JB.01349-07. 18223072 PMC2293202

[6] Bowring J, Neamah MM, Donderis J, Mir-Sanchis I, Alite C, Ciges-Tomas JR, Maiques E, Medmedov I, Marina A, Penadés JR. 2017. Pirating conserved phage mechanisms promotes promiscuous staphylococcal pathogenicity island transfer. Elife 6:e26487. doi:10.7554/eLife.26487. 28826473 PMC5779228

[7] Tormo-Más MA, Donderis J, García-Caballer M, Alt A, Mir-Sanchis I, Marina A, Penadés JR. 2013. Phage dUTPases control transfer of virulence genes by a proto-oncogenic G protein-like mechanism. Mol Cell 49:947–958. doi:10.1016/j.molcel.2012.12.013. 23333307

[8] Frígols B, Quiles-Puchalt N, Mir-Sanchis I, Donderis J, Elena SF, Buckling A, Novick RP, Marina A, Penadés JR. 2015. Virus satellites drive viral evolution and ecology. PLoS Genet 11:e1005609. doi:10.1371/journal.pgen.1005609. 26495848 PMC4619825

[9] Donderis J, Bowring J, Maiques E, Ciges-Tomas JR, Alite C, Mehmedov I, Tormo-Mas MA, Penadés JR, Marina A. 2017. Convergent evolution involving dimeric and trimeric dUTPases in pathogenicity island mobilization. PLoS Pathog 13:e1006581. doi:10.1371/journal.ppat.1006581. 28892519 PMC5608427

[10] Hill RLL, Dokland T. 2016. The type 2 dUTPase of bacteriophage φNM1 initiates mobilization of Staphylococcus aureus bovine pathogenicity island 1. J Mol Biol 428:142–152. doi:10.1016/j.jmb.2015.11.009. 26585401 PMC4738164

[11] Ciges-Tomas JR, Alite C, Humphrey S, Donderis J, Bowring J, Salvatella X, Penadés JR, Marina A. 2019. The structure of a polygamous repressor reveals how phage-inducible chromosomal islands spread in nature. Nat Commun 10:3676. doi:10.1038/s41467-019-11504-2. 31417084 PMC6695447

[12] Vértessy BG, Tóth J. 2009. Keeping uracil out of DNA: physiological role, structure and catalytic mechanism of dUTPases. Acc Chem Res 42:97–106. doi:10.1021/ar800114w. 18837522 PMC2732909

[13] Benedek A, Pölöskei I, Ozohanics O, Vékey K, Vértessy BG. 2018. The Stl repressor from Staphylococcus aureus is an efficient inhibitor of the eukaryotic fruitfly dUTPase. FEBS Open Bio 8:158–167. doi:10.1002/2211-5463.12302. PMC5794464 29435406

[14] Hirmondó R, Szabó JE, Nyíri K, Tarjányi S, Dobrotka P, Tóth J, Vértessy BG. 2015. Cross-species inhibition of dUTPase via the staphylococcal Stl protein perturbs dNTP pool and colony formation in Mycobacterium. DNA Repair (Amst) 30:21–27. doi:10.1016/j.dnarep.2015.03.005. 25841100

[15] Nyíri K, Mertens HDT, Tihanyi B, Nagy GN, Kőhegyi B, Matejka J, Harris MJ, Szabó JE, Papp-Kádár V, Németh-Pongrácz V, Ozohanics O, Vékey K, Svergun DI, Borysik AJ, Vértessy BG. 2018. Structural model of human dUTPase in complex with a novel proteinaceous inhibitor. Sci Rep 8:4326. doi:10.1038/s41598-018-22145-8. 29531348 PMC5847570

[16] Maiques E, Quiles-Puchalt N, Donderis J, Ciges-Tomas JR, Alite C, Bowring JZ, Humphrey S, Penadés JR, Marina A. 2016. Another look at the mechanism involving trimeric dUTPases in Staphylococcus aureus pathogenicity island induction involves novel players in the party. Nucleic Acids Res 44:5457–5469. doi:10.1093/nar/gkw317. 27112567 PMC4914113

[17] Pecsi I, Hirmondo R, Brown AC, Lopata A, Parish T, Vertessy BG, Tóth J. 2012. The dUTPase enzyme is essential in Mycobacterium smegmatis. PLoS One 7:e37461. doi:10.1371/journal.pone.0037461. 22655049 PMC3360063

[18] Szabó JE, Németh V, Papp-Kádár V, Nyíri K, Leveles I, Bendes Á, Zagyva I, Róna G, Pálinkás HL, Besztercei B, Ozohanics O, Vékey K, Liliom K, Tóth J, Vértessy BG. 2014. Highly potent dUTPase inhibition by a bacterial repressor protein reveals a novel mechanism for gene expression control. Nucleic Acids Res 42:11912–11920. doi:10.1093/nar/gku882. 25274731 PMC4231751

[19] Alite C, Humphrey S, Donderis J, Maiques E, Ciges-Tomas JR, Penadés JR, Marina A. 2017. Dissecting the link between the enzymatic activity and the SaPI inducing capacity of the phage 80α dUTPase. Sci Rep 7:11234. doi:10.1038/s41598-017-11234-9. 28894239 PMC5593958

[20] Chan S, Segelke B, Lekin T, Krupka H, Cho US, Kim M, So M, Kim C-Y, Naranjo CM, Rogers YC, Park MS, Waldo GS, Pashkov I, Cascio D, Perry JL, Sawaya MR. 2004. Crystal structure of the Mycobacterium tuberculosis dUTPase: insights into the catalytic mechanism. J Mol Biol 341:503–517. doi:10.1016/j.jmb.2004.06.028. 15276840

[21] Mol CD, Harris JM, McIntosh EM, Tainer JA. 1996. Human dUTP pyrophosphatase: uracil recognition by a β hairpin and active sites formed by three separate subunits. Structure 4:1077–1092. doi:10.1016/S0969-2126(96)00114-1. 8805593

[22] Wang F, Liu C, Wang C, Wang Y, Zang K, Wang X, Liu X, Li S, Li F, Ma Q. 2021. Structural basis of staphylococcal Stl inhibition on a eukaryotic dUTPase. Int J Biol Macromol 184:821–830. doi:10.1016/j.ijbiomac.2021.06.107. 34171258

[23] Takács E, Nagy G, Leveles I, Harmat V, Lopata A, Tóth J, Vértessy BG. 2010. Direct contacts between conserved motifs of different subunits provide major contribution to active site organization in human and mycobacterial dUTPases. FEBS Lett 584:3047–3054. doi:10.1016/j.febslet.2010.05.018. 20493855 PMC2922844

[24] Baldo AM, McClure MA. 1999. Evolution and horizontal transfer of dUTPase-encoding genes in viruses and their hosts. J Virol 73:7710–7721. doi:10.1128/JVI.73.9.7710-7721.1999. 10438861 PMC104298

[25] Tarbouriech N, Buisson M, Seigneurin JM, Cusack S, Burmeister WP. 2005. The monomeric dUTPase from Epstein-Barr virus mimics trimeric dUTPases. Structure 13:1299–1310. doi:10.1016/j.str.2005.06.009. 16154087

[26] Varga B, Barabás O, Kovári J, Tóth J, Hunyadi-Gulyás É, Klement É, Medzihradszky KF, Tölgyesi F, Fidy J, Vértessy BG. 2007. Active site closure facilitates juxtaposition of reactant atoms for initiation of catalysis by human dUTPase. FEBS Lett 581:4783–4788. doi:10.1016/j.febslet.2007.09.005. 17880943

[27] Pegan SD, Tian Y, Sershon V, Mesecar AD. 2010. A universal, fully automated high throughput screening assay for pyrophosphate and phosphate release from enzymatic reactions. Comb Chem High Throughput Screen 13:27–38. doi:10.2174/138620710790218203. 20201823

[28] Vardakou M, Salmon M, Faraldos JA, O’Maille PE. 2014. Comparative analysis and validation of the malachite green assay for the high throughput biochemical characterization of terpene synthases. MethodsX 1:187–196. doi:10.1016/j.mex.2014.08.007. 26150952 PMC4472957

[29] Powell HR, Johnson O, Leslie AGW. 2013. Autoindexing diffraction images with iMosflm. Acta Crystallogr D Biol Crystallogr 69:1195–1203. doi:10.1107/S0907444912048524. 23793145 PMC3689522

[30] Evans PR, Murshudov GN. 2013. How good are my data and what is the resolution? Acta Crystallogr D Biol Crystallogr 69:1204–1214. doi:10.1107/S0907444913000061. 23793146 PMC3689523

[31] Winn MD, Ballard CC, Cowtan KD, Dodson EJ, Emsley P, Evans PR, Keegan RM, Krissinel EB, Leslie AGW, McCoy A, McNicholas SJ, Murshudov GN, Pannu NS, Potterton EA, Powell HR, Read RJ, Vagin A, Wilson KS. 2011. Overview of the CCP4 suite and current developments. Acta Crystallogr D Biol Crystallogr 67:235–242. doi:10.1107/S0907444910045749. 21460441 PMC3069738

[32] McCoy AJ, Grosse-Kunstleve RW, Adams PD, Winn MD, Storoni LC, Read RJ. 2007. Phaser crystallographic software. J Appl Crystallogr 40:658–674. doi:10.1107/S0021889807021206. 19461840 PMC2483472

[33] Murshudov GN, Skubák P, Lebedev AA, Pannu NS, Steiner RA, Nicholls RA, Winn MD, Long F, Vagin AA. 2011. REFMAC5 for the refinement of macromolecular crystal structures. Acta Crystallogr D Biol Crystallogr 67:355–367. doi:10.1107/S0907444911001314. 21460454 PMC3069751

[34] Emsley P, Lohkamp B, Scott WG, Cowtan K. 2010. Features and development of Coot. Acta Crystallogr D Biol Crystallogr 66:486–501. doi:10.1107/S0907444910007493. 20383002 PMC2852313

[35] Krissinel E, Henrick K. 2007. Inference of macromolecular assemblies from crystalline state. J Mol Biol 372:774–797. doi:10.1016/j.jmb.2007.05.022. 17681537

[36] Echols N, Grosse-Kunstleve RW, Afonine PV, Bunkóczi G, Chen VB, Headd JJ, McCoy AJ, Moriarty NW, Read RJ, Richardson DC, Richardson JS, Terwilliger TC, Adams PD. 2012. Graphical tools for macromolecular crystallography in PHENIX. J Appl Crystallogr 45:581–586. doi:10.1107/S0021889812017293. 22675231 PMC3359726

[37] Krieger E, Joo K, Lee J, Lee J, Raman S, Thompson J, Tyka M, Baker D, Karplus K. 2009. Improving physical realism, stereochemistry and side-chain accuracy in homology modeling: four approaches that performed well in CASP8. Proteins 77:114–122. doi:10.1002/prot.22570. 19768677 PMC2922016

[38] Mirdita M, Steinegger M, Söding J. 2019. MMseqs2 desktop and local web server app for fast, interactive sequence searches. Bioinformatics 35:2856–2858. doi:10.1093/bioinformatics/bty1057. 30615063 PMC6691333

[39] Katoh K, Misawa K, Kuma KI, Miyata T. 2002. MAFFT: a novel method for rapid multiple sequence alignment based on fast Fourier transform. Nucleic Acids Res 30:3059–3066. doi:10.1093/nar/gkf436. 12136088 PMC135756

[40] Gouy M, Guindon S, Gascuel O. 2010. SeaView version 4: a multiplatform graphical user interface for sequence alignment and phylogenetic tree building. Mol Biol Evol 27:221–224. doi:10.1093/molbev/msp259. 19854763

[41] Capella-Gutiérrez S, Silla-Martínez JM, Gabaldón T. 2009. trimAl: a tool for automated alignment trimming in large-scale phylogenetic analyses. Bioinformatics 25:1972–1973. doi:10.1093/bioinformatics/btp348. 19505945 PMC2712344

[42] Wheeler TJ, Clements J, Finn RD. 2014. Skylign: a tool for creating informative, interactive logos representing sequence alignments and profile hidden Markov models. BMC Bioinformatics 15:7. doi:10.1186/1471-2105-15-7. 24410852 PMC3893531

