# Supplementary material for: The Bacteriophage–Phage-Inducible Chromosomal Island Arms Race Designs an Interkingdom Inhibitor of dUTPases
Source: Microbiol Spectr. 2023 Jan 9;11(1):e03232-22. doi: 10.1128/spectrum.03232-22 (PMC9927489; doi:10.1128/spectrum.03232-22)
Supplement: Supplemental file 1 — Supplemental material. Download spectrum.03232-22-s0001.pdf, PDF file, 3.6 MB [file spectrum.03232-22-s0001.pdf]

Supplementary data

Bacteriophage-PICI arms race designs an interkingdom inhibitor of dUTPases

Carla Sanz-Frasquet[1], J. Rafael Ciges-Tomas[1], Christian Alite[1], José R. Penadés[2,3*], Alberto Marina[1*].

[1]Instituto de Biomedicina de Valencia (IBV), CSIC and CIBER de Enfermedades Raras (CIBERER), C/ Jaume Roig 11, 46010, Valencia, Spain

[2]Institute of Infection, Immunity and Inflammation, College of Medical, Veterinary and Life Sciences, University of Glasgow, Glasgow, G12 8TA, UK

[3]MRC Centre for Molecular Bacteriology and Infection, Imperial College London, SW7 2AZ, UK.

*Correspondence:
Alberto Marina
Instituto de Biomedicina de Valencia Consejo Superior de Investigaciones Científicas
Email: amarina@ibv.csic.es

José R. Penadés
MRC Centre for Molecular Bacteriology and Infection Imperial College London
Email: j.penades@imperial.ac.uk

CONTENTS

**Supplementary Figures**	page
Figure S1	3
Figure S2	4
Figure S3	5
Figure S4	6
Figure S5	7
Figure S6	8
Figure S7	9
Figure S8	10
Figure S9	11
Figure S10	12
**Supplementary Tables**	
Table S1	13
Table S2	14
Table S3	15
Table S4	16
Table S5	17

Supplementary Figures

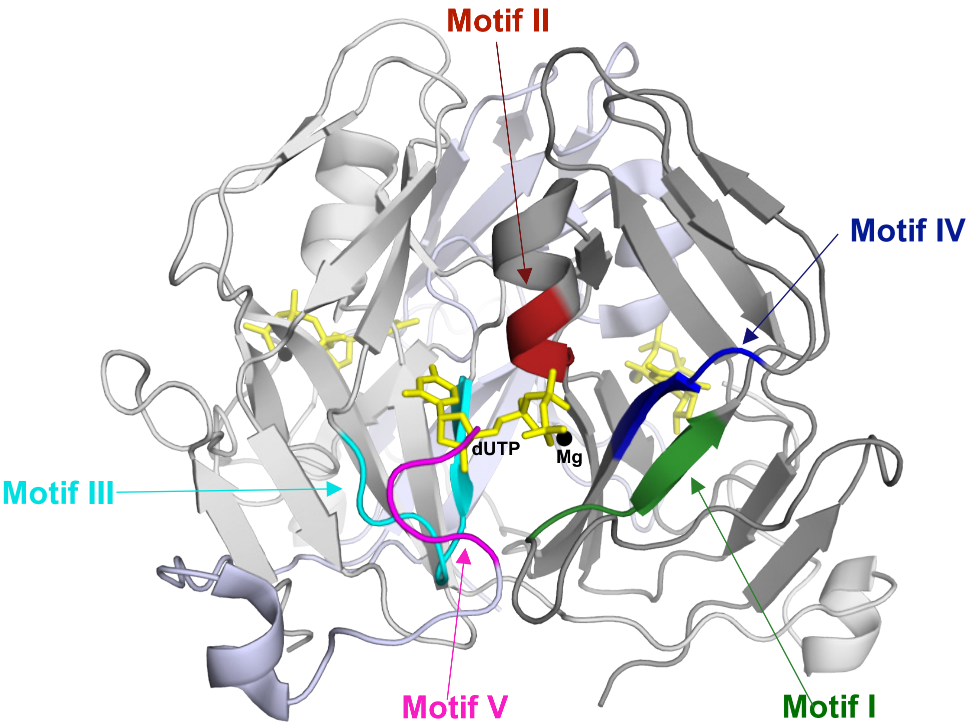

Figure S1. Trimeric Dut catalytic motifs. The five catalytic motifs of trimeric Duts are highlighted for one active center with differ colours in the structure of mDut-dUTP complex. Each Dut monomer is coloured in gray tones. dUTPs are shown in sticks (yellow) and the Mg ion in spheres (black).

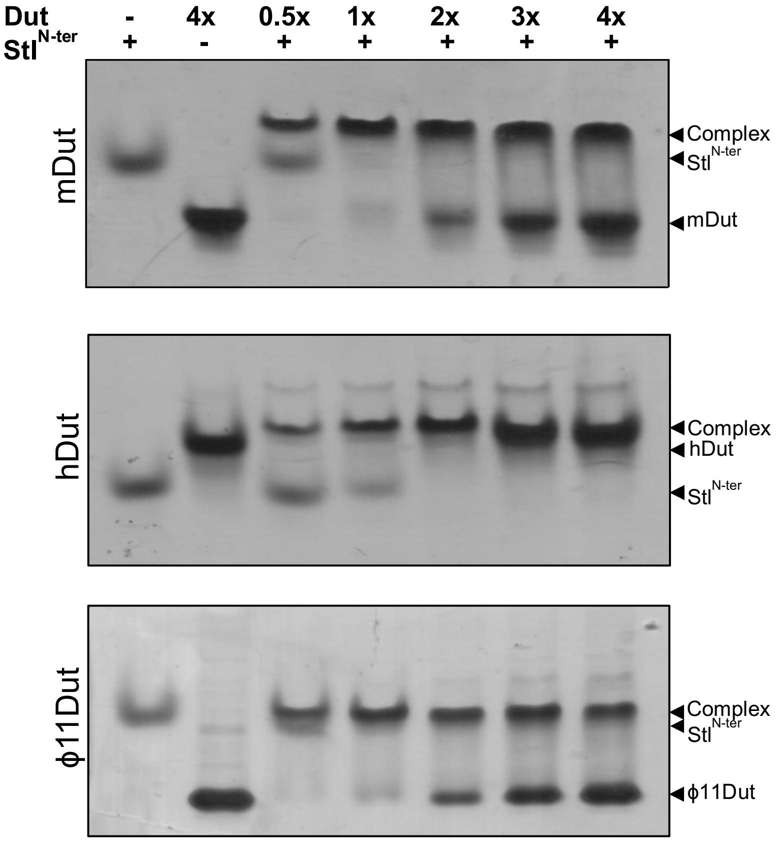

Figure S2. Native-PAGE analysis of $Stl^{N\text{-}ter}$ with mDut, hDut and ϕ11Dut. Increasing molar ratio concentrations of Duts were added to a fix molar concentration of $Stl^{N\text{-}ter}$.

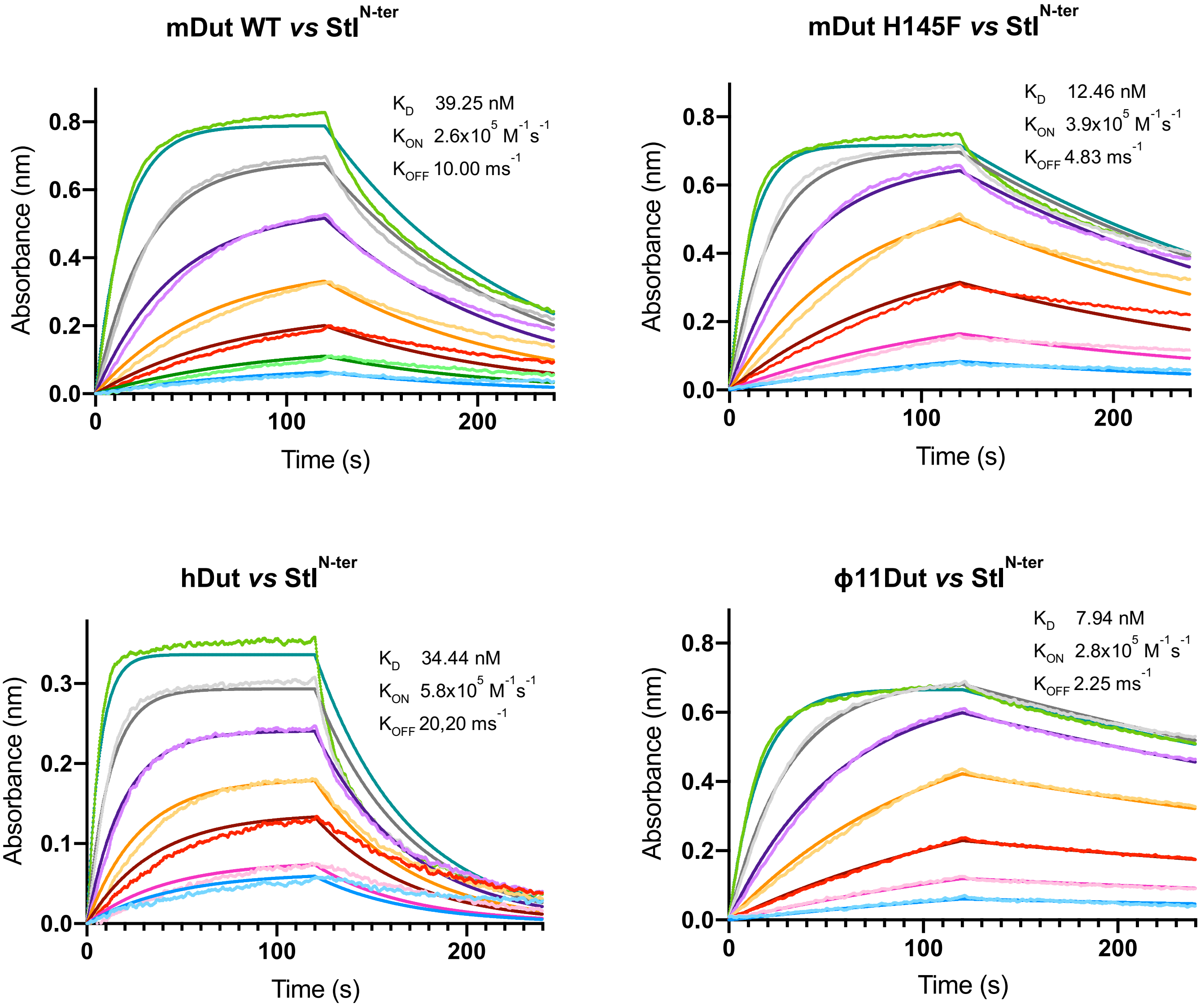

Figure S3. Determination of the binding kinetics of $Stl^{N\text{-}ter}$ with mDut, mDut H145F, hDut and ϕ11Dut by biolayer interferometry. Sensorgrams for representative assays with 7 increasing concentrations of analyte ($Stl^{N\text{-}ter}$) in the steps of association (120 seconds) and dissociation (120 seconds) showing in light colors the experimental data and dark colors the fitting for a 1:1 model. The calculated binding values (K_D, K_{ON} and K_{OFF}) for the representative experiment are shown.

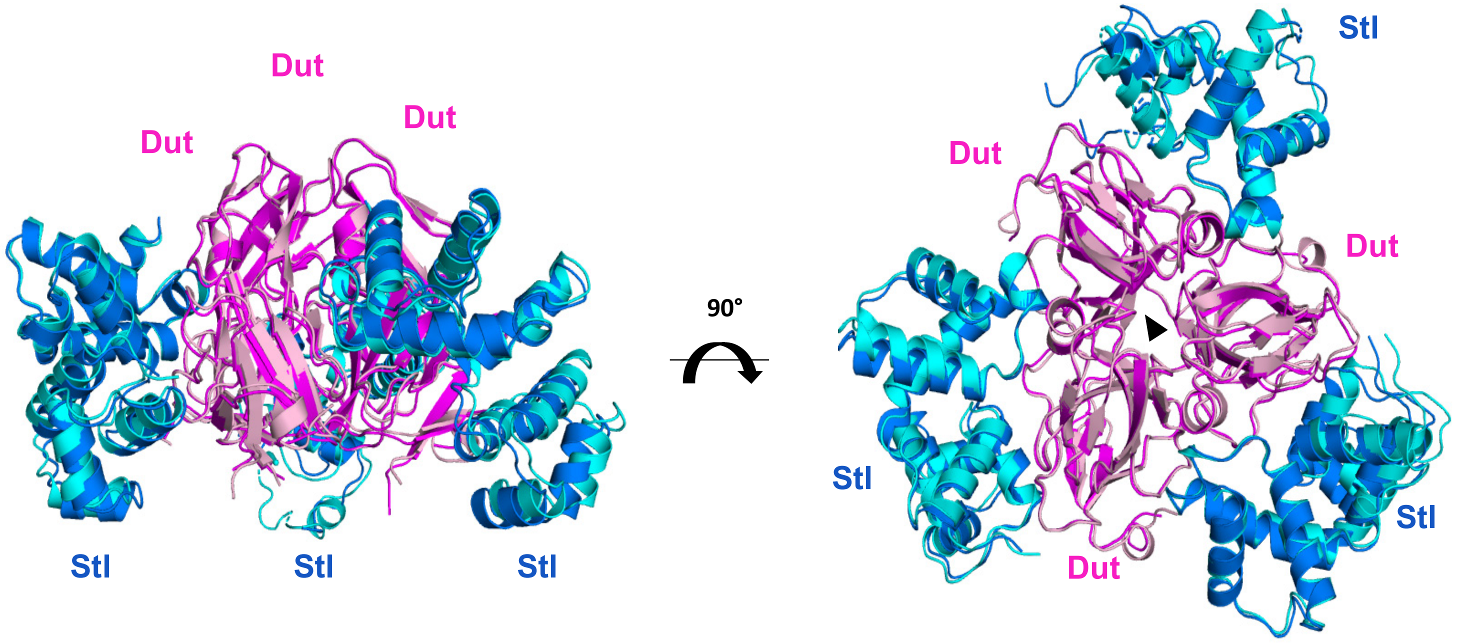

Figure S4. Two orthogonal views of the superposition of the two mDut-Stl$^{N\text{-}ter}$ complexes present in the asymmetric unit of the crystal. One complex is coloured in magenta and cyan, and the second one in lightpink and marine blue for mDut and Stl$^{N\text{-}ter}$, respectively.

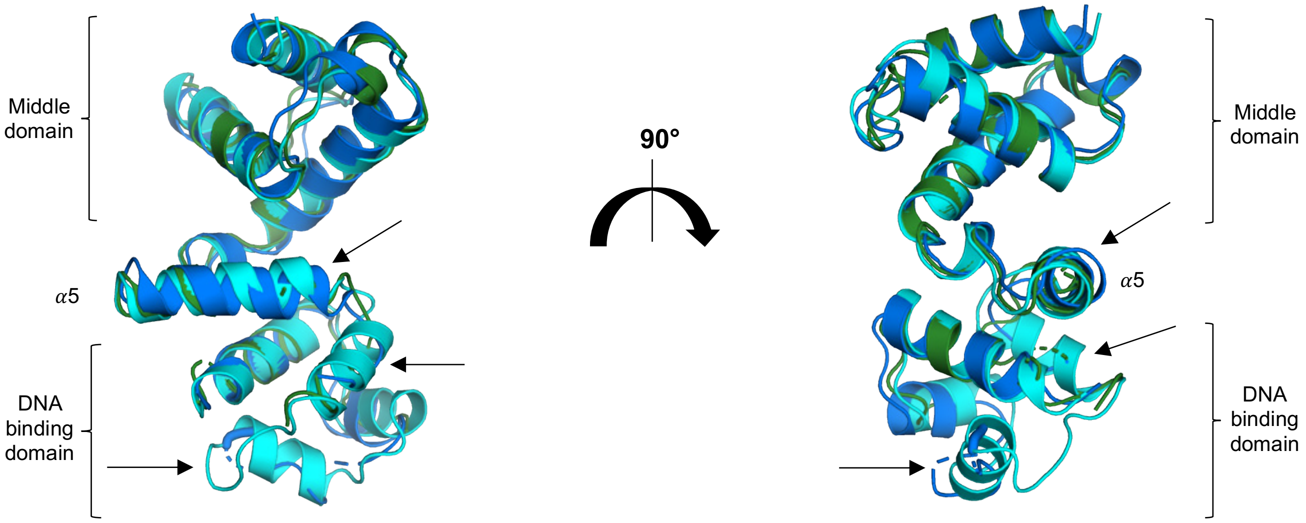

Figure S5. Superimposition of three-dimensional structures of Stl$^{N\text{-}ter}$ monomers present in the mDut-Stl$^{N\text{-}ter}$ complex. The asymmetric unit of the mDut-Stl$^{N\text{-}ter}$ crystal structure contains 2 complexes of trimeric Dut with 3 Stls. In each complex one Stl monomer shows regions of high flexibility. The two monomers with high flexible regions (cyan and green) are superimposed with one representative stable monomer (marine blue) and the regions of flexibility are indicated by arrows. Two orthogonal views are shown.

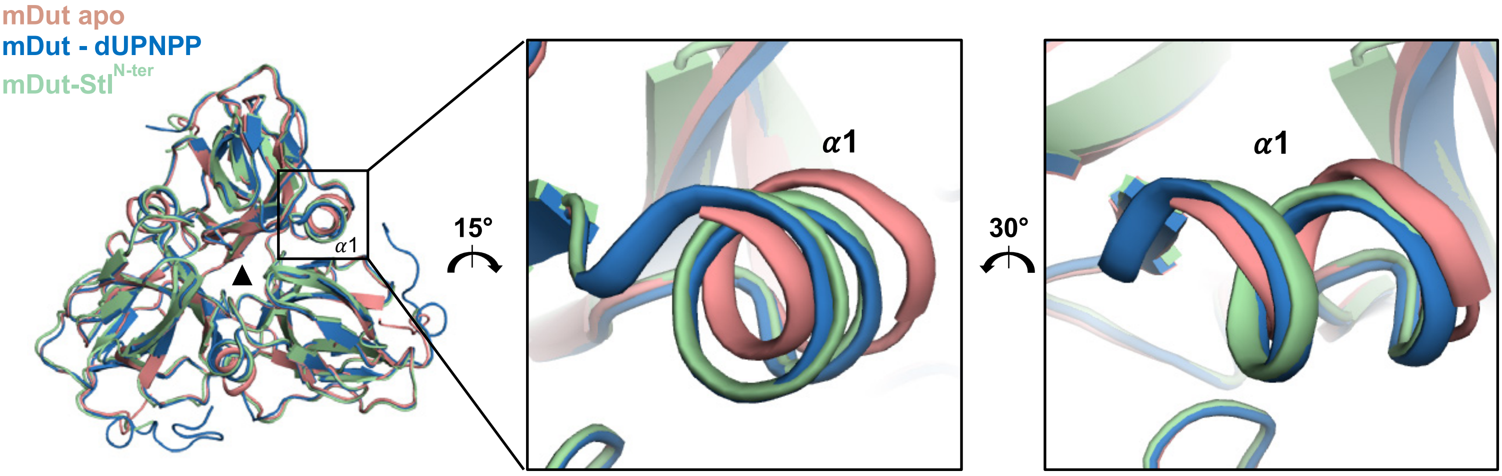

Figure S6. mDut trimer structure in complex with Stl is more similar to the nucleotide-bound than Apo form. Close-view of the mDut helix α1 (residues 65-70) in the superimposition of the apo (salmon), nucleotide-bound (marine blue) and Stl-bound (green) structures shows a loss of the helical topology in the apo state that is retained in the nucleotide and Stl-bound states.

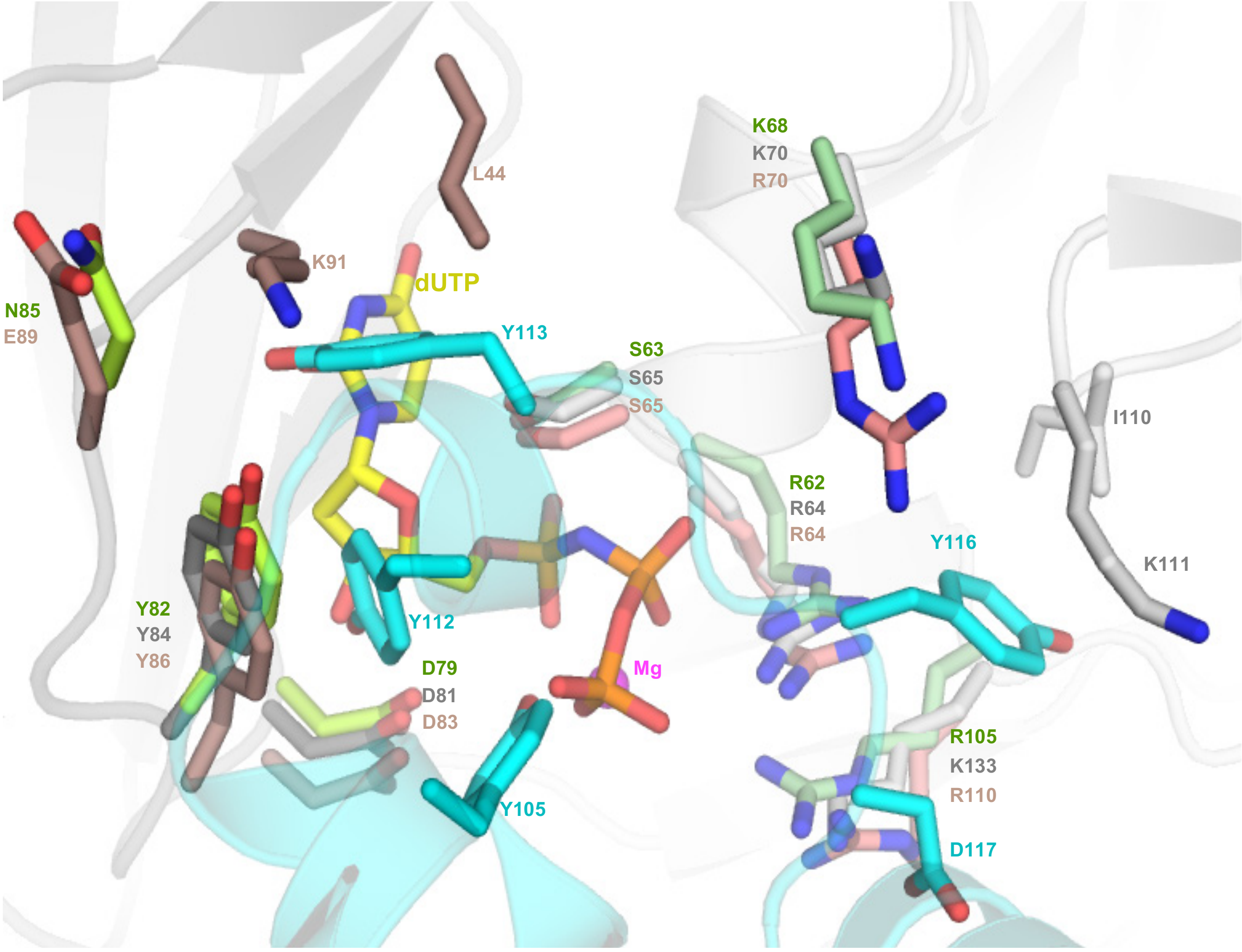

Figure S7. Stl mimics interactions of the dUTP substrate with trimeric Duts. Detail view of the Dut-Stl interaction region for three superimposed Stl complexes with hDut (green), ϕ11Dut (gray) and mDut (brown). A molecule of dUPNPP (substrate analogue) was placed in the active center of the Duts by superimposing the structure of ϕ11Dut – dUPNPP (PDB 4gv8) in the ϕ11Dut-Stl complex and is shown in sticks with carbon atoms in yellow and the Mg ion chelated by the nucleotide is shown as a magenta sphere. Duts and Stl (cyan) interacting residues are shown in stick and labelled.

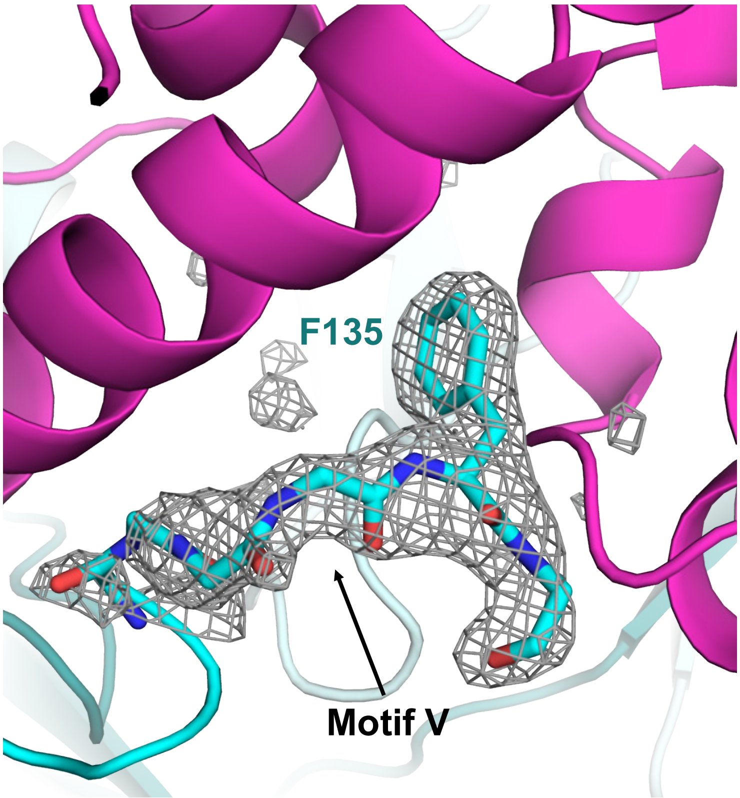

Figure S8. hDut **m**otif V interaction with Stl$^{N\text{-}ter}$. Fo-Fc omit map calculated for the hDut motif V (residues R130-G136) is contoured as a gray mesh at 3 σ level over the final model of this motif represented in stick with carbon atoms coloured in cyan. hDut F135, which projects in a Stl (represent in magenta cartoon) hydrophobic pocket, is labelled.

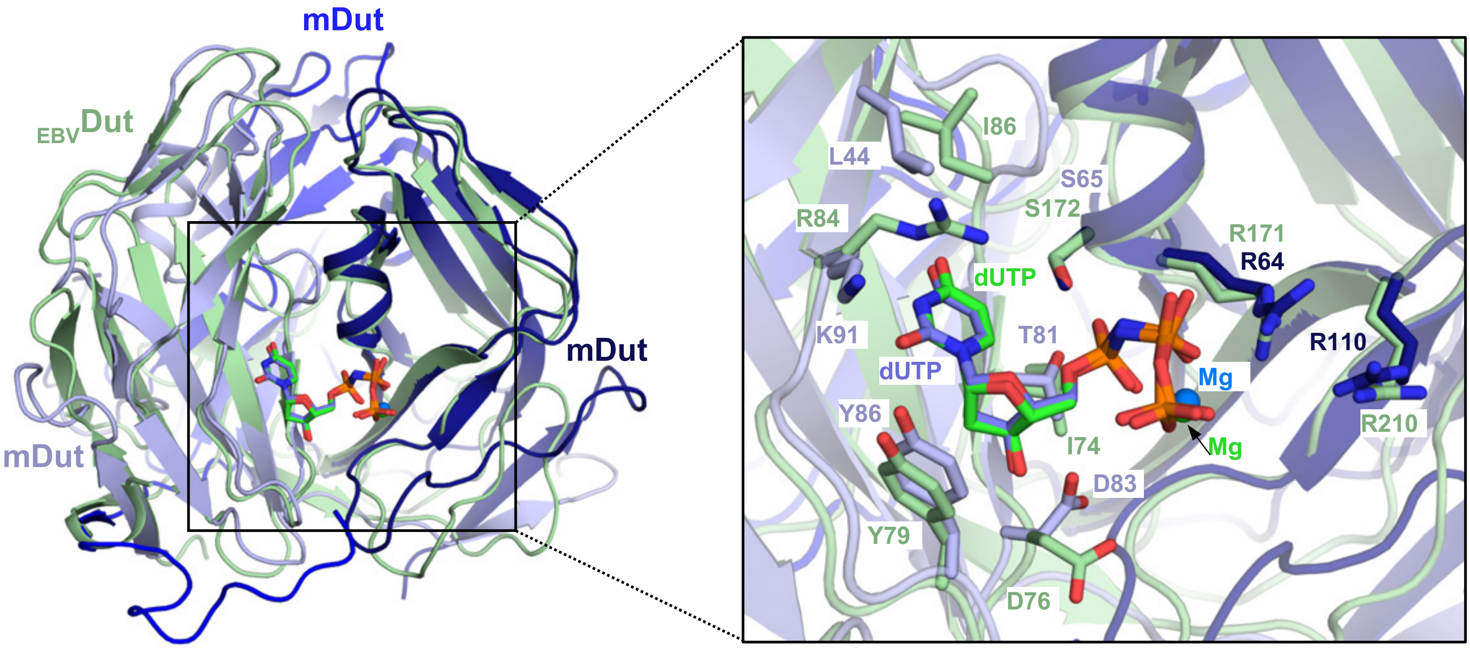

Figure S9. Structural comparison of monomeric and trimeric Duts. Superimposition of the substrate nucleotides on trimeric mDut and monomeric $_{EBV}$Dut highlights the structural conservation of the active centers for both families of Duts. *Left,* the cartoon representation of both molecules (mDut subunits is coloured in a blue tone and $_{EBV}$Dut in green) shows the structural alignment of secondary elements after nucleotide superimposition. *Right*, a close view of the active centers shows the identical disposition of the nucleotides and the chelated Mg ions (n sticks and spheres, respectively) and the conservation of several catalytic residues (represented in sticks with the carbon atoms colored as the respective molecules) in both types of Duts

A

gccatcatcatcatcatcac **gag aat ctt tac ttt caa gga atg cag ttg cgc ttt gca cgt ttg tcc gaa cat gct acg gcc ccc aca aga ggt tca gcg cgt gcg gct ggt tat gac ctg tat tca gct tat gac tac acc atc cct ccg atg gag aag gct gta gtt aag act gat att cag atc gct ttg cca tcg ggt tgc tat ggc cgg gtt gcg ccg cgt agt ggg tta gcg gca aag cac ttc att gat gtg gga gct ggt gtg att gac gaa gat tat cgg gga aac gtg gga gtc gtc tta ttc aac ttc ggt aag gag aaa ttt gaa gta aag aaa ggt gac cgg ata gca cag tta atc tgc gaa aga ata ttt tat cct gaa att gag gaa gtc cag gcg ctt gac gat acc gag aga ggg tct gga ggg ttt ggc tcg acg ggg aaa aat taa** ctagcataaccccttggggc

B

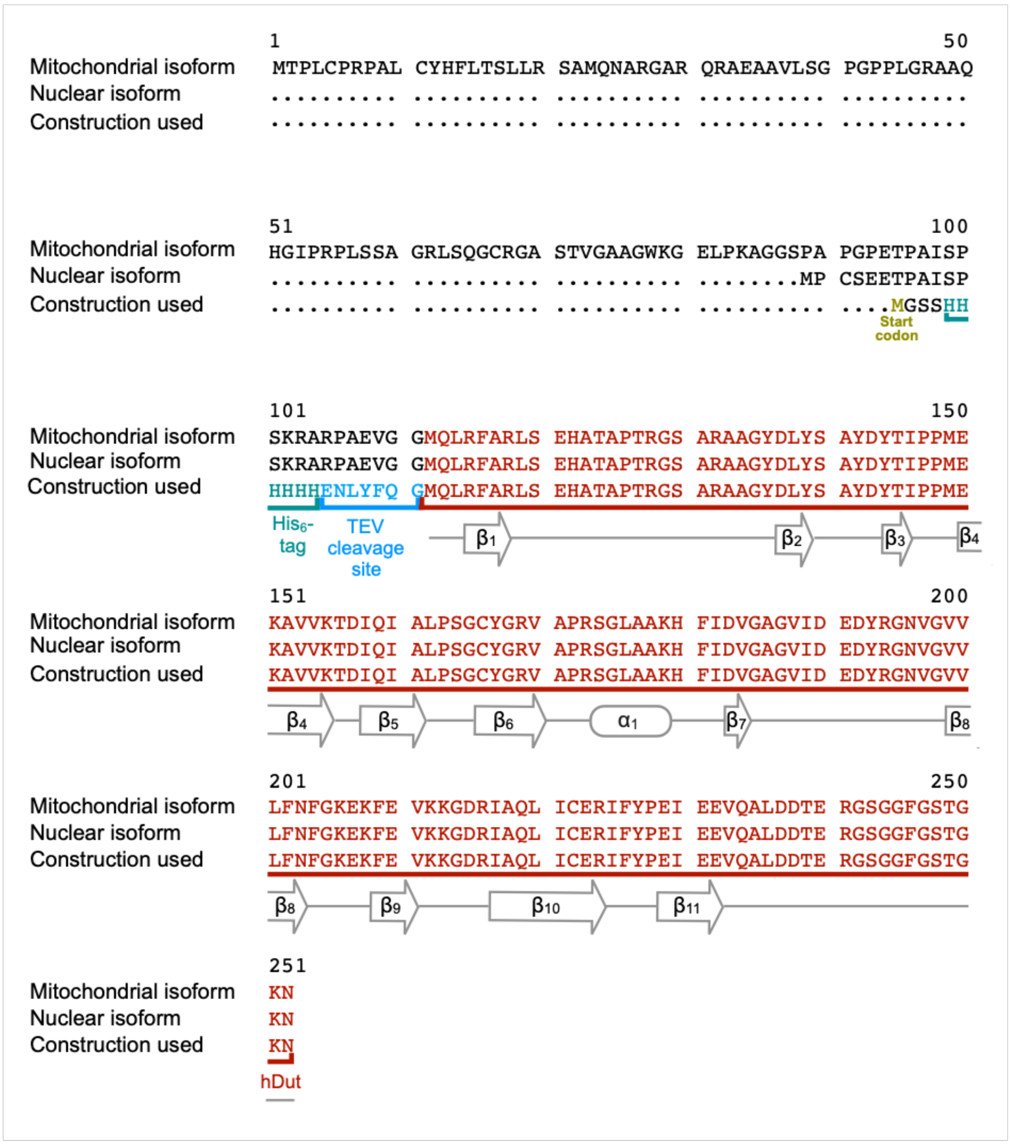

Figure S10. (A) Optimized DNA sequence of the human Dut used in this work (bold) flanked by the overhangs required for cloning (in blue). (B) Alignment of the protein sequences of the two human isoforms of Dut (mitochondrial and nuclear) and the protein sequence used in this work. The construction used involves a common part of both isoforms, and includes a His_6-tag at the N-terminus followed by a TEV protease cleavage site. The secondary structure is also indicated. Alignment source: *Multalin*.

Supplementary Tables

Table S1. Oligonucleotides used in this study.

Plasmid	Oligonucleotides	Sequence 5' – 3'
pET28a	pET28a-Fw	CTAGCATAACCCCTTGGG
	pET28a-Rv	GTGATGATGATGATGATGG
	hDut-Fw	GCCATCATCATCATCATCAC
	hDut-Rv	GCCCCAAGGGGTTATGCTAG
pETNKI 1.1	mDut-Fw	CAGGGACCCGGTGTGTCGACCACTCTGGCGATC
	mDut-Rv	CGAGGAGAAGCCCGGTTATCACAAACTCGCATGTCCGCC
	mDutH145F-Fw	CGACGGTGGCTTTGGTTCCTCCGGCGG
	mDutH145F-Rv	CCGCGGGATGTCGAGGCC

Table S2. Intermolecular interactions for mDut-Stl$^{N\text{-}ter}$ complex.

**Stl$^{N\text{-}ter}$**			**mDut**			
Structural element	Residue	Atom type	Structural element	Residue	Atom type	Distance (Å)
α4	55 Val	CG2	β1	9 Arg	NH2	3.8
		CG1	Lβ8-End	126 Glu	OE2	3.5
	56 Asn	ND2			O	2.8
	59 Glu	OE1	Lβ1β2	18 Ser	OG	3.0
α7	102 Asn	OD1		21 His	NE2	3.6
	106 Tyr	OH	Lβ1β2		CB	2.9
				24 Asp	OD2	2.8
		O		87 Arg	N	2.7
				88 Gly		3.7
	107 Asn					3.9
α8	109 Gly	N		86 Tyr	OH	3.5
		O	β6	91 Lys	NZ	3.7
	110 Asp	OD1				3.1
	112 Tyr	CG	Lβ1β2	86 Tyr	CE1	3.3
		OH	β5	81 Thr	O	3.8
			Lβ5β6	83 Asp	N	3.1
	113 Tyr	O	α1	66 Gly		3.5
			Lβ4α1	65 Ser	O	3.7
			α1	69 Thr	OG1	2.6
		CB	β3	44 Leu	CD2	3.8
		OH	β6	91 Lys	N	2.8
			Lβ5β6	89 Glu	O	3.5
		CE2		86 Tyr	OH	3.5
	114 Ser	O	α1	70 Arg	NE	3.7
α9	116 Tyr	OH				3.1
		CZ		67 Leu	CD1	3.3
		O	Lβ4α1	64 Arg	NH2	2.8
	117 Asp	OD2	β7	110 Arg	NE	3.4
α10	152 Leu	O	α1	70 Arg	NH1	3.4

Table S3. Intermolecular interactions for hDut-Stl$^{N\text{-}ter}$ complex.

**Stl$^{N\text{-}ter}$**			**hDut**			
Structural element	Residue	Atom type	Structural element	Residue	Atom type	Distance (Å)
α4	56 Asn	OD1	β8	121 Glu	N	2.9
	59 Glu	CD		119 Ile	CG2	3.7
	66 Gly	O	Lβ11-End	136 Gly	N	3.2
Lα4α5	67 Ile	CG1		135 Phe	CB	3.9
	68 Pro	CD			CE1	3.4
α5	70 Tyr	CB	Lβ1β2	16 Thr	CB	3.9
		CE1		18 Gly	CA	3.9
	74 Arg	NH1	β2	28 Tyr	OH	2.9
α7	98 Tyr	CD2	Lβ11-End	135 Phe	CZ	3.3
	99 Ser	CB		133 Gly	C	3.5
				134 Gly	CA	3.8
	102 Asn	ND2		130 Arg	O	2.9
	106 Tyr	CD1	Lβ5β6	81 Asp	CB	3.9
		O		83 Arg	N	2.8
				84 Gly	N	3.7
	107 Asn	OD1	Lβ11-End	127 Asp	OD1	3.2
		ND2		131 Gly	O	3.1
α8	109 Gly	N	Lβ5-β6	82 Tyr	OH	3.5
	112 Tyr	CG			CE1	3.2
		OH	β5	77 Val	O	3.5
				79 Asp	N	3.0
	113 Tyr	CE1	Lβ5-β6	82 Tyr	OH	3.7
				85 Asn	O	3.9
		CZ	β6	87 Gly	CA	3.5
		OH			O	2.6
					N	2.7
		C	α1	64 Gly	CA	3.9
	114 Ser	OG		68 Lys	CG	3.5
α9	116 Tyr	O	Lβ4α1	62 Arg	NH1	2.6
		OH	Lβ6-β7	104 Asp	OD1	3.6
				105 Arg	N	2.8
	117 Asp	OD2			NH1	3.0

Table S4. Intermolecular interactions for EBVDut-Stl$^{N\text{-}ter}$ model.

**Stl$^{N\text{-}ter}$**		**EBVDut**	
Structural element	Residue	Structural element	Residue
α4	55 Val	L β12-β13	118 Glu
	56 Asn		117 Glu
	59 Glu		115 Gln
α5	74 Arg	L β19-β20	210 Arg
α7	102 Asn	L β12-β13	128 Tyr
	105 Tyr	L β9-β10	76 Asp
	106 Tyr		78 Gly
			80 Thr
	110 Asp		84 Arg
	112 Tyr		79 Tyr
		β9	74 Iso
	113 Tyr	α1	172 Ser
α9	116 Tyr	L β16-α1	171 Arg
		α1	173 Gly
			174 Leu
			177 Gln
	117 Asp	L β19-β20	210 Arg

Table S5. interactions of Dut recognition residues of Stl$^{N\text{-}ter}$ in the complexes with mDut, hDut, ϕ11Dut and $_{EBV}$Dut.

Stl$^{N\text{-}ter}$	ϕ11DUT	mDUT	hDUT	$_{EBV}$Dut
55 Val	149 Gln	126 Glu	121 Glu	118 Glu
56 Asn				117 Glu
59 Glu		18 Ser		115 Gln
			119 Ile	
66 Gly			136 Gly	
67 Ile			135 Phe	
68 Pro				
70 Tyr	18 Glu		16 Thr	
	20 Asn		18 Gly	
74 Arg	18 Glu			
			28 Tyr	
77 Asp	15 Arg			
98 Tyr	20 Asn			
			135 Phe	
99 Ser			133 Gly	
			134 Gly	
102 Asn	20 Asn			
		21 His		128 Tyr
			130 Arg	
106 Tyr	21 His	21 His		
	24 Asp	24 Asp		
			81 Asp	78 Gly
	85 His	87 Arg	83 Arg	80 Thr
		88 Gly	84 Gly	
107 Asn			127 Asp	
			131 Gly	
109 Gly	84 Tyr	86 Tyr	82 Tyr	
		91 Lys		
110 Asp				84 Arg
112 Tyr	84 Tyr	86 Tyr	82 Tyr	79 Tyr
	79 Lys	81 Thr	77 Val	74 Ile
	81 Asp	83 Asp	79 Asp	
113 Tyr	66 Gly	66 Gly		
	65 Ser	65 Ser		
	69 Ser	69 Thr		
		44 Leu		
	89 Gly	91 Lys	87 Gly	
		89 Glu	85 Asn	
		86 Tyr	82 Tyr	
			64 Gly	
114 Ser	70 Lys	70 Arg	68 Lys	
116 Tyr				173 Gly
		67 Leu		
	64 Arg	64 Arg	62 Arg	171 Arg
	110 Ile	Absence of motif VI		
	111 Lys			
			104 Asp	
117 Asp	133 Lys	110 Arg	105 Arg	210 Arg
152 Leu	70 Lys	70 Arg		
